# Progress of fracture mapping technology based on CT three-dimensional reconstruction

**DOI:** 10.3389/fbioe.2024.1471470

**Published:** 2024-11-06

**Authors:** Jichao Liu, Ziyan Zhang, Ji Qu, Chengdong Piao

**Affiliations:** Department of Orthopedics, The Second Hospital of Jilin University, Changchun, China

**Keywords:** fracture mapping, heat map, morphology, fractures, classification, three-dimensional

## Abstract

Fracture Mapping is a new technology developed in recent years. This technology visually representing the morphology of fractures by overlaying fracture lines from multiple fracture models onto a standard model through three-dimensional reconstruction. Fracture mapping has been widely used in acetabular fracture, proximal humerus fractures, Pilon fracture, tibial plateau fractures, and so on. This technology provides a new research method for the diagnosis, classification, treatment selection, internal fixation design, and statistical analysis of common fracture sites. In addition, the fracture map can also provide a theoretical basis for the establishment of a biomechanical standardized fracture model. Herein, we reviewed various methods and the most advanced techniques for fracture mapping, and to discuss the issues existing in fracture mapping techniques, which will help in designing future studies that are closer to the ideal. Moreover, we outlined the fracture morphology features of fractures in various parts of the body, and discuss the implications of these fracture mapping studies for fracture treatment, thereby providing reference for research and clinical decision-making on bone and joint injuries to improve patient prognosis.

## 1 Introduction

X-rays are commonly utilized as the fundamental auxiliary examinations for diagnosing fractures, and many traditional fracture classifications are based on X-rays (Fu et al., 2019). With the progress in radiology, computed tomography (CT) and three-dimensional (3D) CT have become extensively employed in clinical practice to offer precise evaluation and diagnosis for fractures (Cho et al., 2018). Over the past 2 decades, there has been a development in fracture mapping technology aimed at visualizing fracture patterns and extracting statistical characteristics from extensive clinical data sets. The fracture mapping visually representing the morphology of fractures by overlaying fracture lines from multiple fracture models onto a standard model through 3D reconstruction (Molenaars et al., 2015; Hadad et al., 2018; Mitchell et al., 2019; Yao X. et al., 2022; Chao et al., 2023; Ren et al., 2023b). By analyzing a sufficient number of samples, information regarding the prevalence, morphology, and frequency of fracture lines can be directly observed on the standard template.

The emergence of fracture mapping technology visually demonstrates the morphological characteristics of fractures, providing a new research method for fracture diagnosis, classification, treatment selection, internal fixation design, statistical analysis of common fracture sites, and the establishment of standardized fracture models. Over the past 20 years, fracture mapping has been widely used in scapular fractures, pelvic fractures, acetabular fractures, Pilon fractures, tibial plateau fractures, and so on. In particular, in the past 5 years, there has been a surge in research on fracture mapping, with a growing interest in the study of fracture maps.

There are several reasons why it is necessary to write a review on fracture mapping. First, there is no review article on fracture mapping. Second, with the advancement of technology, the research methods of fracture mapping have become more advanced and accurate, and the methods are diverse. Therefore, it is necessary to review these methods and elaborate on the advantages and disadvantages of these methods to provide reference for future research. Third, fracture mapping has been widely used for various types of fractures. Therefore, it is crucial to review the fracture morphology of these fractures, which is particularly important for clinicians making clinical decisions and engineers designing improved implants.

For the above reasons, we conducted a literature review to achieve the following three objectives:

1. To introduce various methods and the most advanced techniques for fracture mapping, and to discuss the issues existing in fracture mapping techniques, which will help in designing future studies that are closer to the ideal.
2. To systematically summarize the fracture morphology features of fractures in various anatomical regions of the body, and to introduce the significance of the findings of these fracture mapping studies for the treatment of fractures.
3. To identify the aspects that need to be improved in fracture mapping research.

## 2 Literature search strategy

We conducted a systematic search across the Web of Science, Scopus, and MEDLINE (via PubMed), databases, covering the earliest available records up to May 2024, using specific search criteria:

(“map*” OR “morphology”) AND (“fracture*”)

Only mapping studies on human fractures that were written in English and published in peer-reviewed journals were included. Literature reviews and case studies were excluded.

**FIGURE 1:**
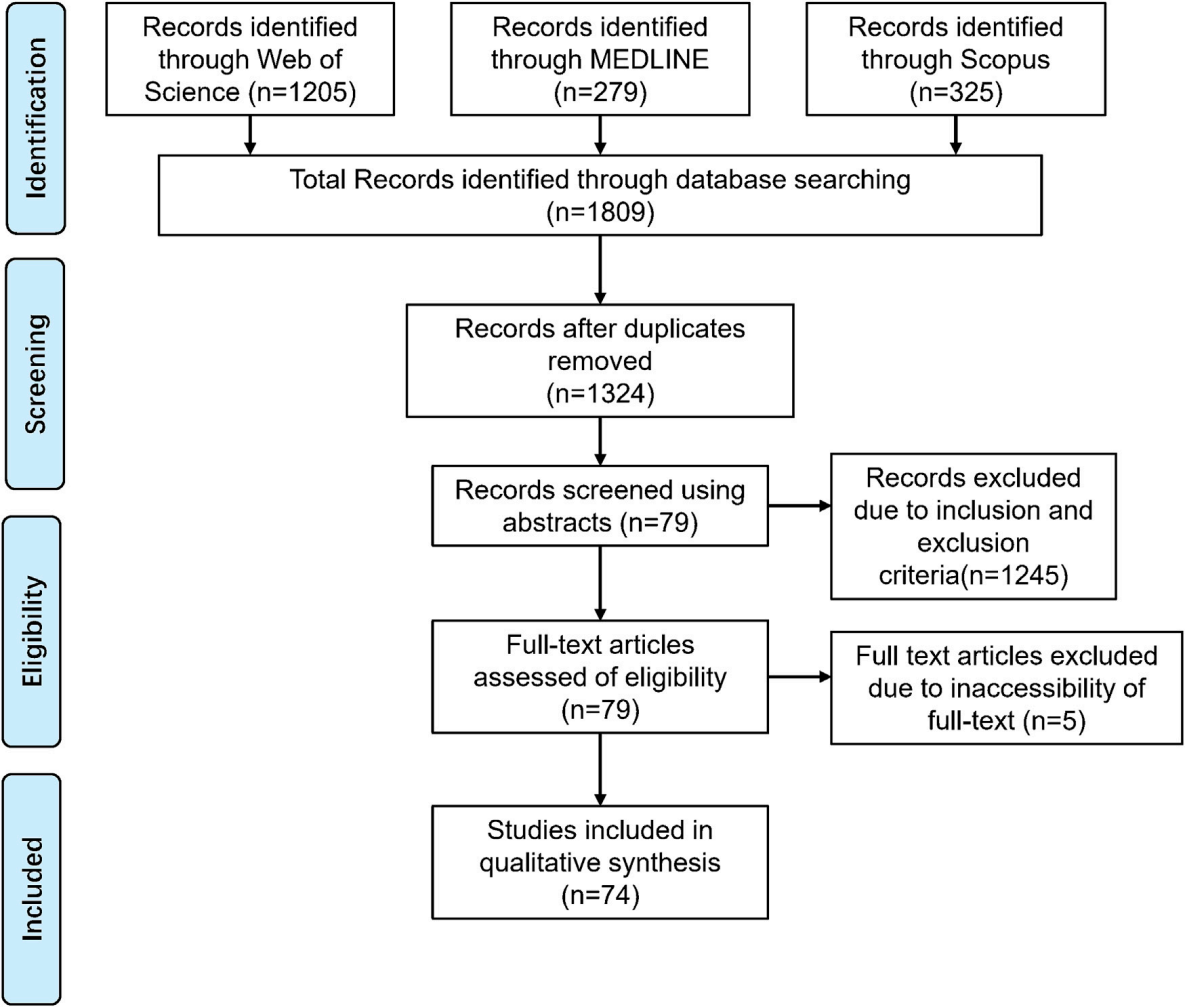
Search and screening flow chat.

**FIGURE 2:**
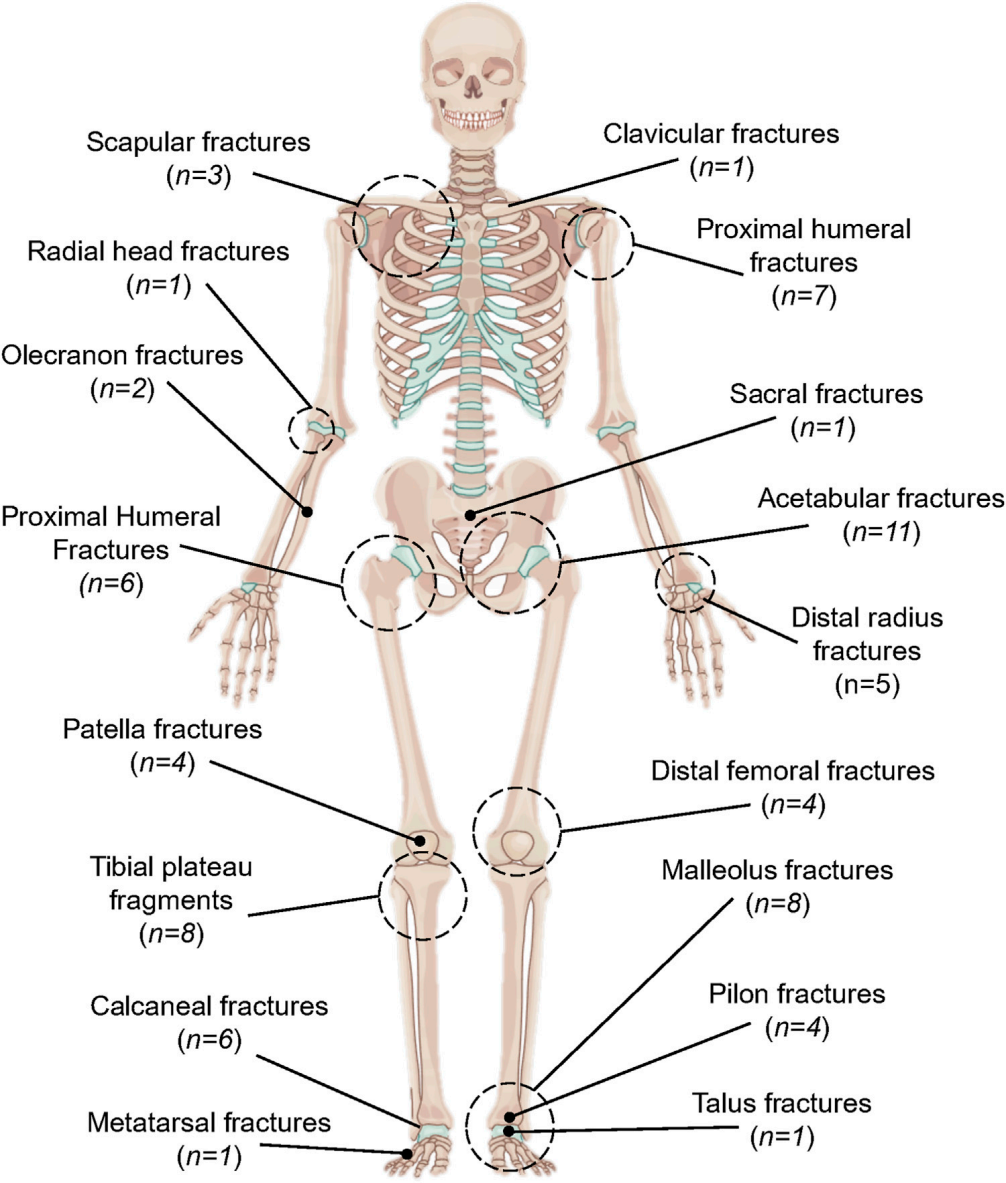
Type and number of fractures involved in the included studies.

**FIGURE 3:**
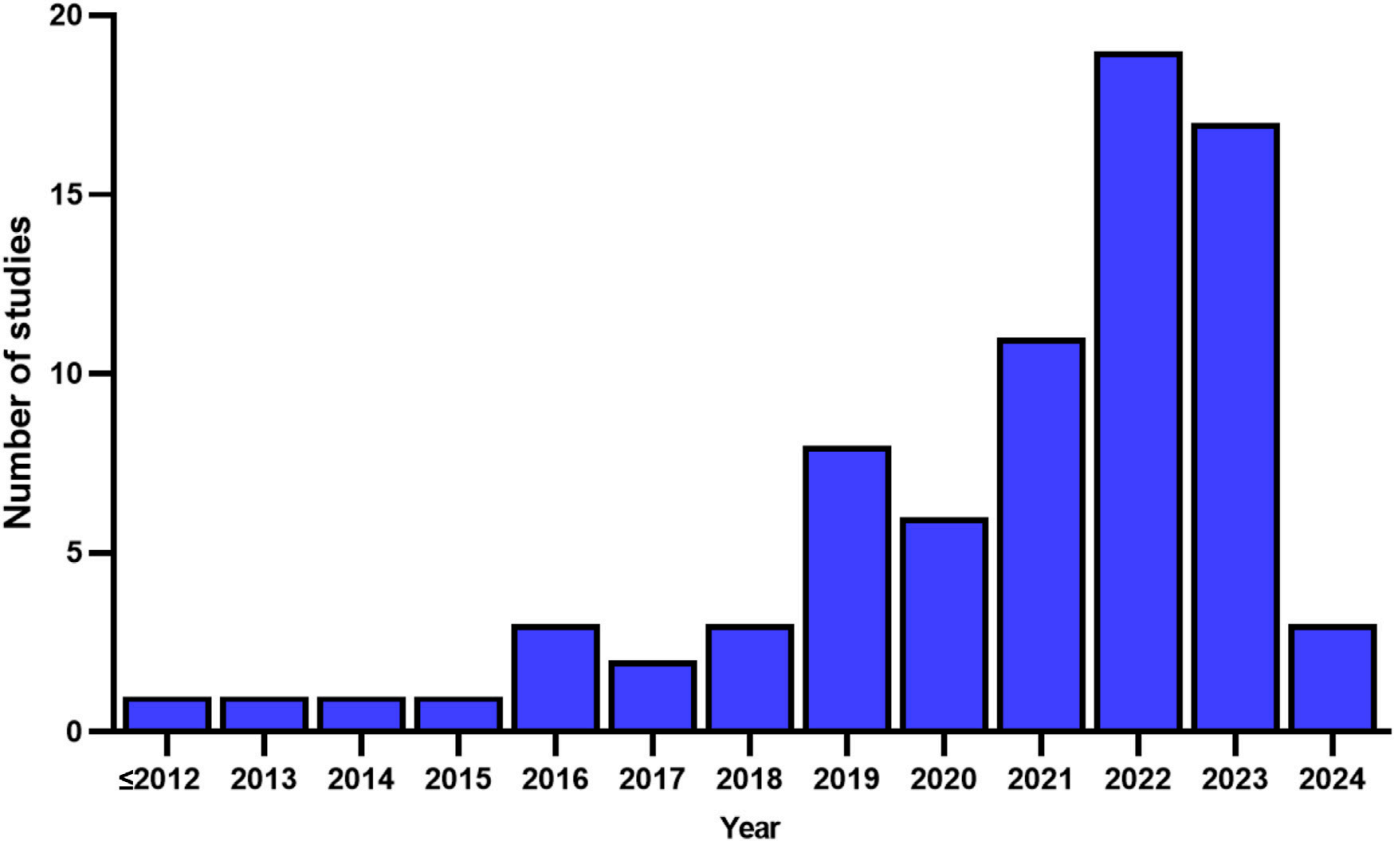
Annual publication volume of studies on fracture mapping.

A total of 1,809 articles were retrieved (Figure 1). The titles and abstracts of these studies were screened to determine their eligibility. After removing duplicates and applying inclusion criteria, only 79 articles were found to be relevant. Among these 79 studies, full texts for 5 could not be obtained, and the abstracts alone did not provide sufficient information for a thorough review. Ultimately, 74 articles were included. Based on the search results, we found that scholars appear to have an increasing interest in mapping techniques, with a significant rise in the number of mapping studies in recent years (Figure 2). The types and numbers of fractures studied using mapping techniques are detailed in Figure 3.

## 3 The fracture mapping

### 3.1 2D fracture mapping

#### 3.1.1 Superimposition of 3D reconstructed images for fracture mapping

**FIGURE 4:**
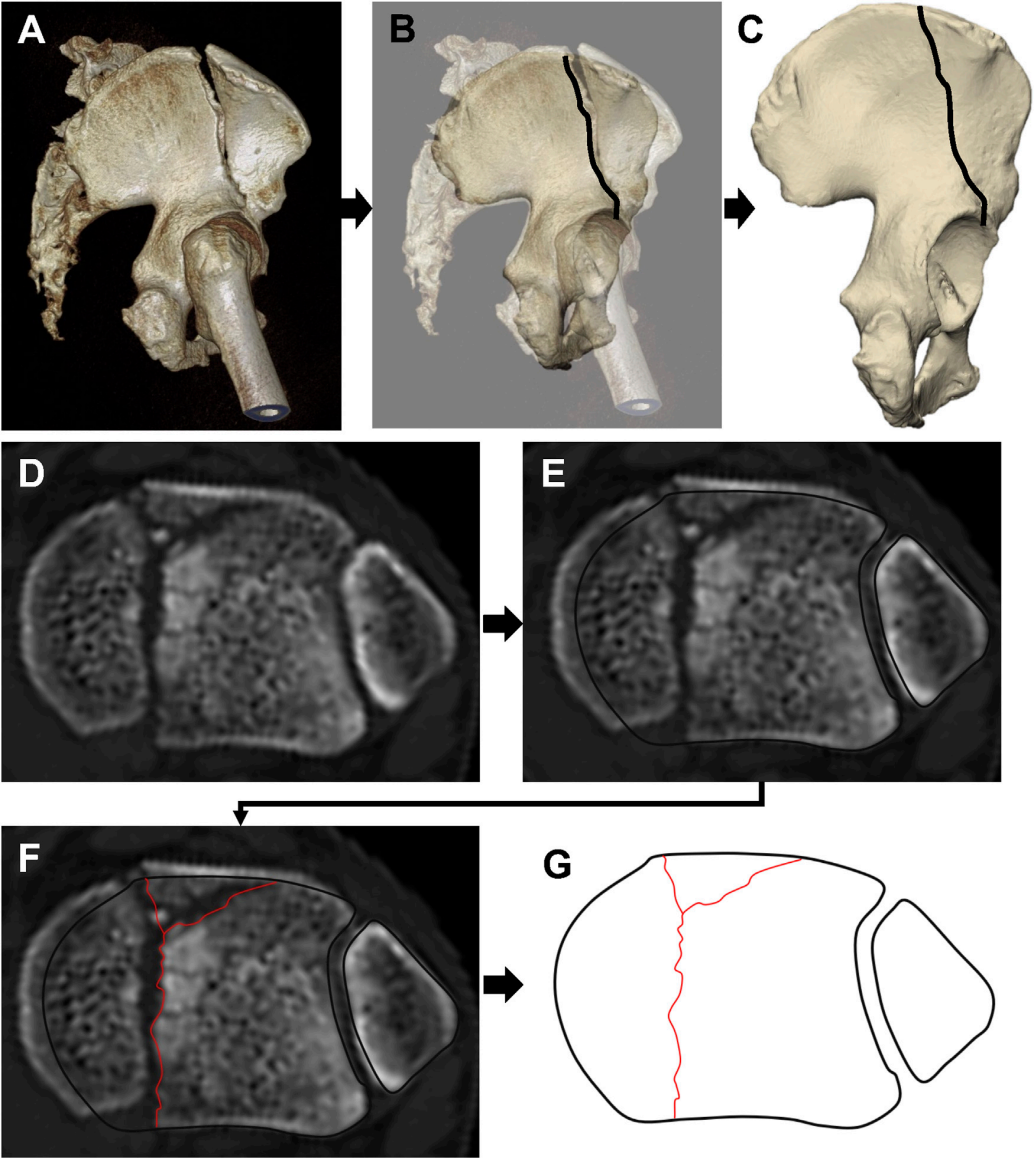
A series of images showing the process of 2D fracture mapping, starting with **(A)** a computed tomography image, **(B)** mapping the fracture line onto a matching standard template, and **(C)** ending with the fracture line mapped onto the standard template. **(D–G)** Superimposition of CT transverse images for fracture mapping: **(D)** CT cross-sectional image 3 mm below the unreduced tibial articular surface; **(E)** Alignment of the CT image with the template; **(F)** Fracture line transcription onto the standard template; **(G)** Completed fracture line transcription.

In 2009, the orthopedic team at the University of Minnesota introduced the concept of fracture mapping and reported the method of two-dimensional (2D) fracture mapping for the first time. The procedure involves the following steps (Figures 4A–C): Initially, 3D reconstruction images of all patients are obtained, either directly from patient examination data or by reconstructing the fracture model using software such as OsiriX (Pixmeo, Bernex, Switzerland) or Mimics (Materialise, Leuven, Belgium). The optimal viewing angle to best display the fracture line is determined for each perspective. Subsequently, in software like Fireworks (Macromedia, San Francisco, California) or Photoshop, the bone marks are aligned through rotation and scaling, and the fracture images are registered with a standard template. The fracture image is then superimposed onto the template, and a fracture line is drawn. Ultimately, a fracture map is generated by overlaying these lines, which directly illustrates the morphological distribution and areas prone to fractures. However, this technique has several limitations: firstly, it does not involve reducing the fracture fragments, which can lead to inaccuracies in describing the fracture line if there is significant displacement or rotation of the fragments; secondly, although it is based on 3D reconstruction of CT data, it only displays the fracture line distribution from a specific 2D perspective, essentially categorizing it as a 2D fracture mapping technique.

#### 3.1.2 Superimposition of CT transverse images for fracture mapping

In 2013, Cole et al. (2013) enhanced the existing technology for 2D fracture mapping. To more accurately depict the characteristics of intra-articular fractures, they shifted from relying on a specific perspective of 3D reconstruction to utilizing the fracture lines from CT scans taken 3 mm below the articular surface (Figures 4D–G). These images were then overlaid onto a standard template in Adobe Fireworks or Photoshop to create the final 2D fracture map.

The difference between the methods in Section 3.1.1 and Section 3.1.2 is as follows: the method described in Section 3.1.1 uses 3D reconstructed images to create the fracture map, whereas the method in Section 3.1.2 utilizes the original CT cross-sectional images (CT cross-sections 3 mm below the articular surface) for fracture mapping. However, similar to the method in Section 3.1.1, the method in Section 3.1.2 does not reduce the fracture fragments and only shows the distribution of fracture lines in a 2D view.

#### 3.1.3 Fracture reduction and mapping

**FIGURE 5:**
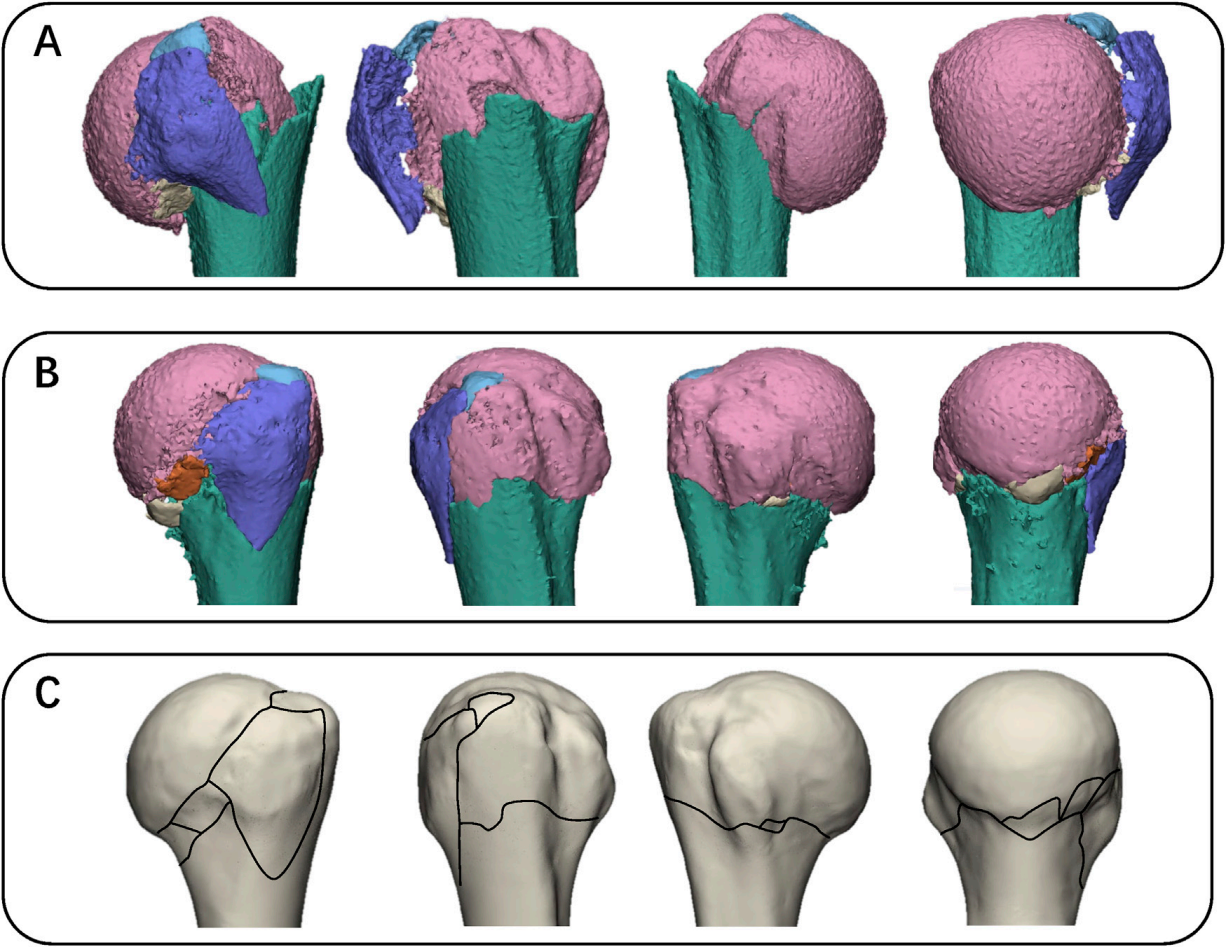
Images illustrating the fracture mapping method. **(A)** Each fragment was reconstructed. **(B)** Then reduce the fractured fragments and demonstrate with a specific perspective. **(C)** Fracture lines are manually transcribed onto a two-dimensional template based on their relationship with anatomical landmarks (distance, orientation).

In 2014, Mellema et al. (2014) further refined the 2D fracture mapping technique. Unlike previous iterations, this method involved reducing the fracture fragments prior to mapping (Figure 5). 1) In the initial phase, the “Paint Effect” and “Threshold Paint” tools in the 3D Slicer software are employed to manually annotate bone structures on axial, sagittal, and coronal CT images Figure 5. This process is followed by the generation of a 3D polygonal mesh reconstruction. 2) Subsequently, the model was imported into Rhinoceros (McNeel, Seattle, WA, United States) for fragments reduction. In addition, Mimics and 3-Matic (Materialise, Leuven, Belgium) are capable of obtaining three-dimensional mesh reconstruction images from the same perspective as the standard template: Initially, the Mimics software is employed to reconstruct the mask of the fracture model, utilizing the “Edit Mask” function to separate the fracture fragments; Subsequently, the fracture model is imported into 3-Matic, where the “Interactive Translate” and “Interactive Rotate” functions are utilized to reposition the fracture and align the standard model with the fracture model. 3) Finally, after acquiring the 3D mesh reconstruction images from the same perspective as the standard template, these images were imported into Fireworks (Macromedia Inc., San Francisco). Following the accurate alignment of the images with the two-dimensional template, the fracture lines were delineated.

### 3.2 3D fracture mapping

Compared to the 2D fracture mapping, 3D fracture mapping provides greater accuracy, intuition, and comprehension. The 3D fracture mapping involves the complete superimposition of a 3D fracture model onto a 3D standard model, representing an overlap of the models in 3D space. This differs from the 2D fracture mapping, which merely overlays images. The completed fracture lines can be visualized from any perspective. Various methods exist for 3D fracture mapping, each sharing similar principles but differing in specific operations. The mapping process generally consists of three steps. Initially, the 3D model of the fracture is reconstructed, and the fracture fragments are separated. Subsequently, the fracture is reduced, normalized, and resized to optimally match the standard model. Ultimately, the normalized fracture model is precisely overlaid with the standard model, and the fracture line of the fracture model is meticulously transcribed to the standard model.

#### 3.2.1 3D mapping with mimics and geomagic

**FIGURE 6:**
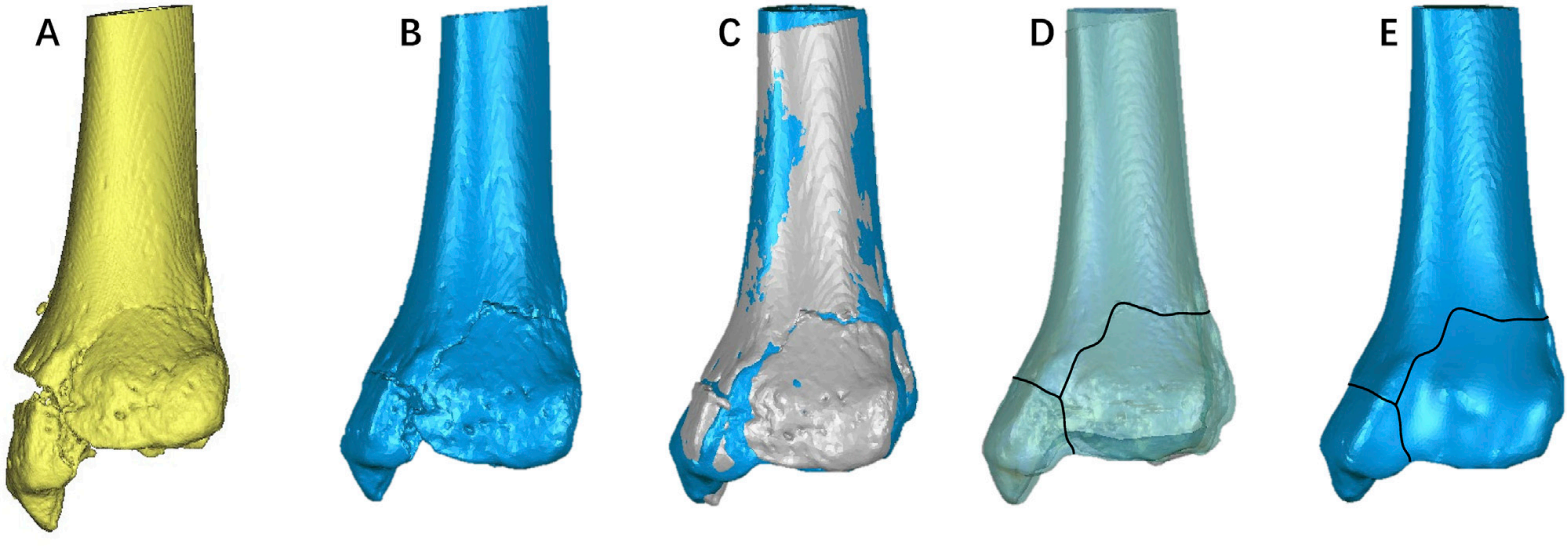
The process of fracture mapping using Mimics and Geomagic. **(A)** Reconstruction of the fracture model in Mimics; **(B)** Importation of the reconstructed fracture model into Geomagic, followed by the segmentation of the fracture model into individual fracture fragments; **(C)** Utilization of Geomagic’s best-fit algorithm to assist in identifying the optimal overlapping position, employing the contralateral side as a template for the reduction of fracture fragments; **(D, E)** With the aid of transparent mode, the fracture line is transferred onto the three-dimensional model of the contralateral side.

In May 2017, Dugarte et al. (2018) introduced the 3D fracture mapping technique for the first time through a controlled study comparing 2D and 3D maps of scapula fractures. The process involves the following steps: First, the fracture model is reconstructed in Mimcs (Figure 6A). Subsequently, the reconstructed fracture model is imported into Geomagic (3D Systems, Rock Hill, SC), where the “crease angle” tool is employed to segment the fracture model into individual fracture fragments (Figure 6B). Thereafter, best-fit algorithm of Geomagic is utilized to assist in identifying the optimal overlapping position, with the contralateral side serving as a template for the reduction of the fracture fragments (Figures 6C,D). Finally, with the aid of the transparent mode, the fracture line is transferred onto the three-dimensional model of the contralateral side (Figure 6E).

#### 3.2.2 3D mapping with mimics and 3-matic

**FIGURE 7:**
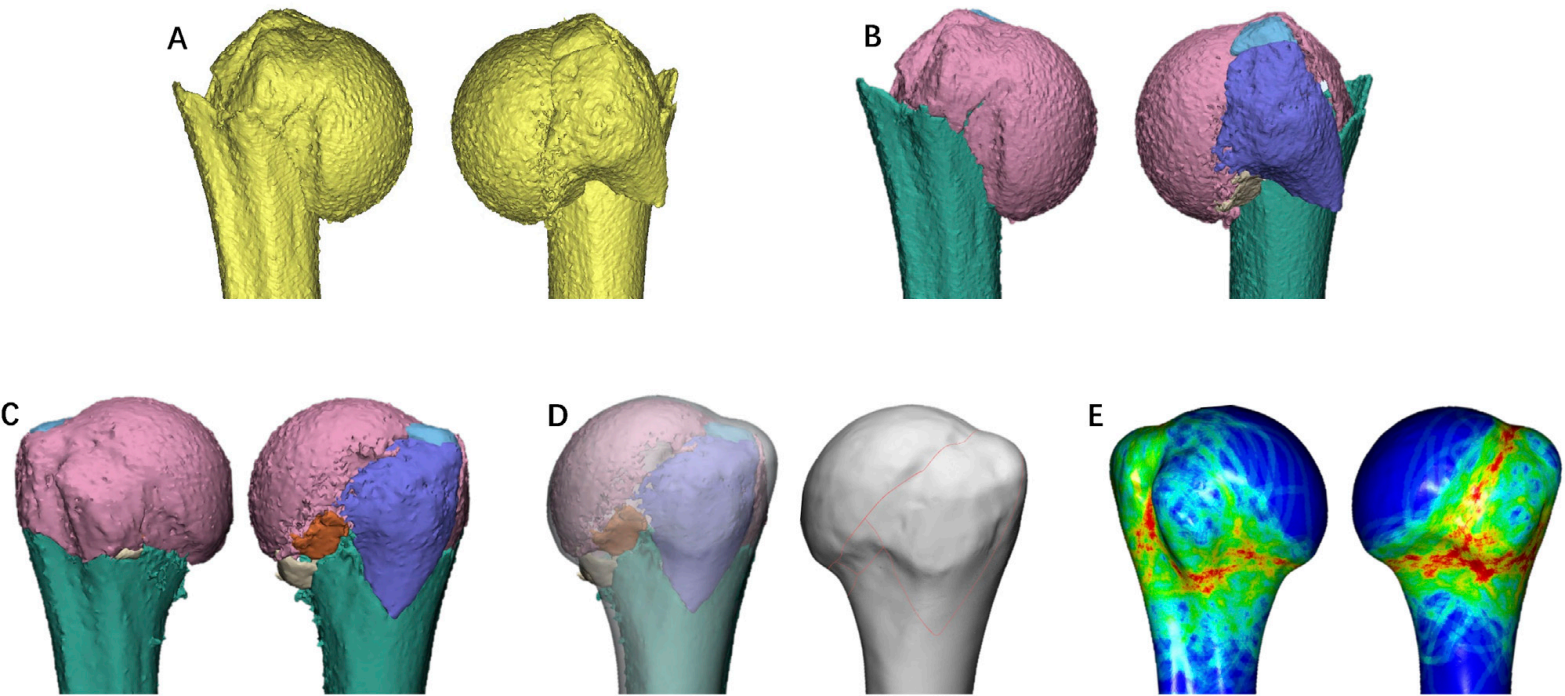
Representative images of the steps in the method used for three-dimensional mapping of proximal humerus fractures. In this example of a proximal humerus fractures, each fracture fragment is reconstructed **(A)**, segmented **(B)**, and virtually reduced **(C)**. Adjust the size of the fracture model and the transparency of the standard model, and transcribe the fracture line onto the standard model **(D)**. Generate a fracture heatmap based on the density of fracture lines **(E)**.

In 2017, Xie et al. (2017) introduced a novel method for 3D mapping of Hoffa fractures, elaborating on the methodology in the appendix of their study. Although this method shares similar principles with the approach by Dugarte et al. (2018), the operational techniques differ. The process of the fracture mapping method described by Xie et al. is as follows (Figure 7): First, the mask of fracture model is reconstructed using Mimics, and the “Edit Mask” function is employed to separate the fracture fragments. Subsequently, the fracture model is imported into 3-Matic, where the “Interactive Translate” and “Interactive Rotate” functions are utilized to realign the fracture and standard models. Finally, the “Transparency” of the standard model is adjusted to “High,” and the “Curve” tool is used to delineate the fracture line on the standard model.

Notably, the virtual reassembly of the fragments deviates from the software’s fitting algorithm, relying instead on manual manipulations such as translation and rotation, enhancing both the universality and accuracy of the fracture reduction. A significant advantage of this approach is that both Mimics and 3-Matic are products of Materialise, facilitating seamless software integration and model importation. Furthermore, this method has standardized steps that have set a precedent for subsequent 3D fracture mapping protocols.

#### 3.2.3 3D mapping with 3D slicer, artec studio and rhinocero

In 2020, Turow et al. (2020) reported on the fracture morphology of scaphoid fractures. Their methodology involved several steps: initially, reverse modeling of the fracture model was performed using 3D Slicer; subsequently, fracture reduction was completed using Artec Studio (Autodesk Inc., San Rafael, CA, United States), followed by overlapping and aligning the fracture model with the standard model; finally, a 3D fracture map was created using Rhinoceros. This has become one of the most commonly used methods in subsequent studies on fracture mapping.

#### 3.2.4 Semi-automated of 3D mapping

In addition to this, a semi-automated method for 3D fracture mapping was reported in 2023 by Mys et al. (2023) Firstly, Matlab R2021b (The Mathworks Ltd., Natick, MA, United States) and C++ were used to identify separated fragments and semi-automatically separate incompletely separated fragments. This workflow is capable of efficiently processing large amounts of CT data. Manual corrections were performed in Amira (Thermo Fisher Scientific, Waltham, MA, United States). The boundary line of each fracture fragment was then identified semi-automatically. The fracture fragments were then reset. The fracture line of each restored fragment was automatically projected onto the standard model by nearest point matching. The semi-automated mapping of fractures significantly enhances the efficiency of the mapping process.

### 3.3 Fracture heat map

In terms of visualization technology, the data representing fracture line distribution density is integrated into the standard model. The density of the fracture lines is illustrated using a color gradient, transitioning from hot to cold colors to provide a more intuitive display of their distribution (Figure 7E). This approach is referred to as heat map technology. Mellema et al. (2014) were the pioneers in applying this technology to 2D fracture maps, specifically analyzing the ulnar coronoid process.

The process of creating a heat map involves three main steps: uniform sampling of fracture lines, assigning density values to the areas surrounding each sampled point, and generating a legend that displays the spatial density distribution of the fracture lines. Despite the apparent simplicity of these steps, the software operation required is complex.

#### 3.3.1 The 2D fracture heat map

First, the image of the fracture map is imported into MATLAB, and the ginput command is utilized to convert the fracture lines into (x, y) coordinates. Subsequently, the point coordinates are exported to Excel and re-imported into MATLAB. Based on these coordinates, the data density is calculated by executing a MATLAB script file, with the results displayed in the form of a heatmap. The density of each point is computed by summing the weighted inverse square distances of other points (Mellema et al., 2014; Hadad et al., 2018). Additionally, it has been reported by some scholars that the external lighting function in Photoshop can be utilized to create a heat map as well.

#### 3.3.2 The 3D fracture heat map

The principle underpinning 3D fracture heat mapping is akin to that of 2D fracture heat mapping, the key distinction being that the former necessitates 3D spatial coordinates. Zeng et al. (2022) reported a method for 3D fracture heat mapping. After delineating the fracture lines in 3-Matic, these lines were subsequently imported into AutoCAD (Autodesk Inc., San Rafael, CA, United States). The fracture line data was then extracted within the AutoCAD software, with a consistent spacing of 0.1 mm, in the form of (x, y, z) coordinates (Zeng et al., 2022). This dataset was then imported into Originlab (OriginLab, Hampton, MA, United States) for the generation of a heat map of the fracture line.

The integration of fracture heat mapping into the E−3D software (Central South University, Changsha, China) has proven beneficial. The procedure for generating a fracture heat map using this software is straightforward and efficient. Initially, the fracture line is delineated in 3-matic and subsequently exported in txt format. This file is then imported into E−3D, where the heat map is produced via the Fracture Line Analysis module. Due to its user-friendly interface, E−3D software has gained popularity for creating fracture heat maps. In addition, Amira (Thermo Fisher Scientific, Waltham, MA, USA) can also generate fracture thermograms, as reported by Mys et al. (2023).

## 4 Overview of fracture morphology

### 4.1 Acetabular fracture

Acetabular fractures are relatively rare, with an incidence of 3 per 100,000 individuals annually (Laird and Keating, 2005) Most acetabular fractures result from high-energy trauma and frequently cause damage to adjacent organ systems (Alvarez-Nebreda et al., 2023). Among the studies reviewed, acetabular fractures were the most frequently examined, with 11 articles focusing on this topic (Table 1). This is likely due to the fact that acetabular fractures pose significant challenges in orthopedic trauma, even for experienced surgeons. Judet et al. (1964) and Butler et al. (2021) have classified acetabular fractures into five basic patterns (posterior wall, posterior column, anterior wall, anterior column, and transverse) and five associated patterns (posterior wall plus posterior column, posterior wall with transverse, anterior plus posterior hemitransverse, T-shaped, and both columns). These classification patterns continue to be widely used in the field today.

**TABLE 1:** Fracture map study of acetabular fractures.

Study	Fractures	Participant Characteristics	2D/3D	Software	Reduction of fractures	Reconstruction of fracture	Heat map
(Yang et al., 2018)	Quadrilateral plate fractures	Gender: 164 male, 68 female Age: overall mean, 43 ± 12y	2D	3D Slicer Fireworks	No	No	No
(Yang et al., 2019a)	Both-column acetabular fractures	Gender: 43 male, 28 female Age: overall mean, 41 ± 14y	2D	3D Slicer Fireworks	No	No	No
(Yang et al., 2019b)	Quadrilateral plate fractures	Gender: 144 male, 64 female Age: overall mean, 43 y	2D	3D Slicer Fireworks	No	No	No
(Zhao et al., 2021)	Acetabular posterior wall fractures	Gender: 45 male, 6 female Age: overall mean, 46 ± 16y	2D	Photoshop	No	No	No
(Ye et al., 2021)	T-shaped acetabular fracture	Gender: 39 male, 17 female Age: overall mean,48.9 ± 12y	3D	Mimics 3-matic E−3D	Yes	Yes	Yes
(Li et al., 2022)	Transverse acetabular fractures	Gender: 32 male, 17 female Age: overall mean,42 ± 13y	3D	Mimics 3-matic E−3D	Yes	Yes	Yes
(Tian et al., 2022)	Complex acetabular fractures	Group A: Gender: 39 male, 6 female Age: overall mean,47.6 ± 13.5y Group B: Gender:64 male, 5 female Age: overall mean,43.0 ± 12.3y	3D	Mimics 3-matic	Yes	Yes	No
(Sinan et al., 2022)	Acetabular fractures	Gender: 47 male, 20 female Age: overall mean,45.2 ± 17y	2D	3D Slicer Photoshop	No	No	Yes
(Yin et al., 2022)	Both-column acetabular fractures	Gender: 55 male, 23female Age:overall mean,49 ± 14y	3D	Mimics 3-matic	No	No	No
(Ye et al., 2022a)	Both-column acetabular fractures	Gender: 81 male, 19 female Age:overall mean,52y	3D	Mimics 3-matic	Yes	Yes	No
(Ye et al., 2023a)	Both-column acetabular fractures	Gender: 81 male, 19 female Age:overall mean,52y	3D	Mimics 3-matic	Yes	Yes	No

#### 4.1.1 Acetabular quadrilateral plate fractures

**FIGURE 8:**
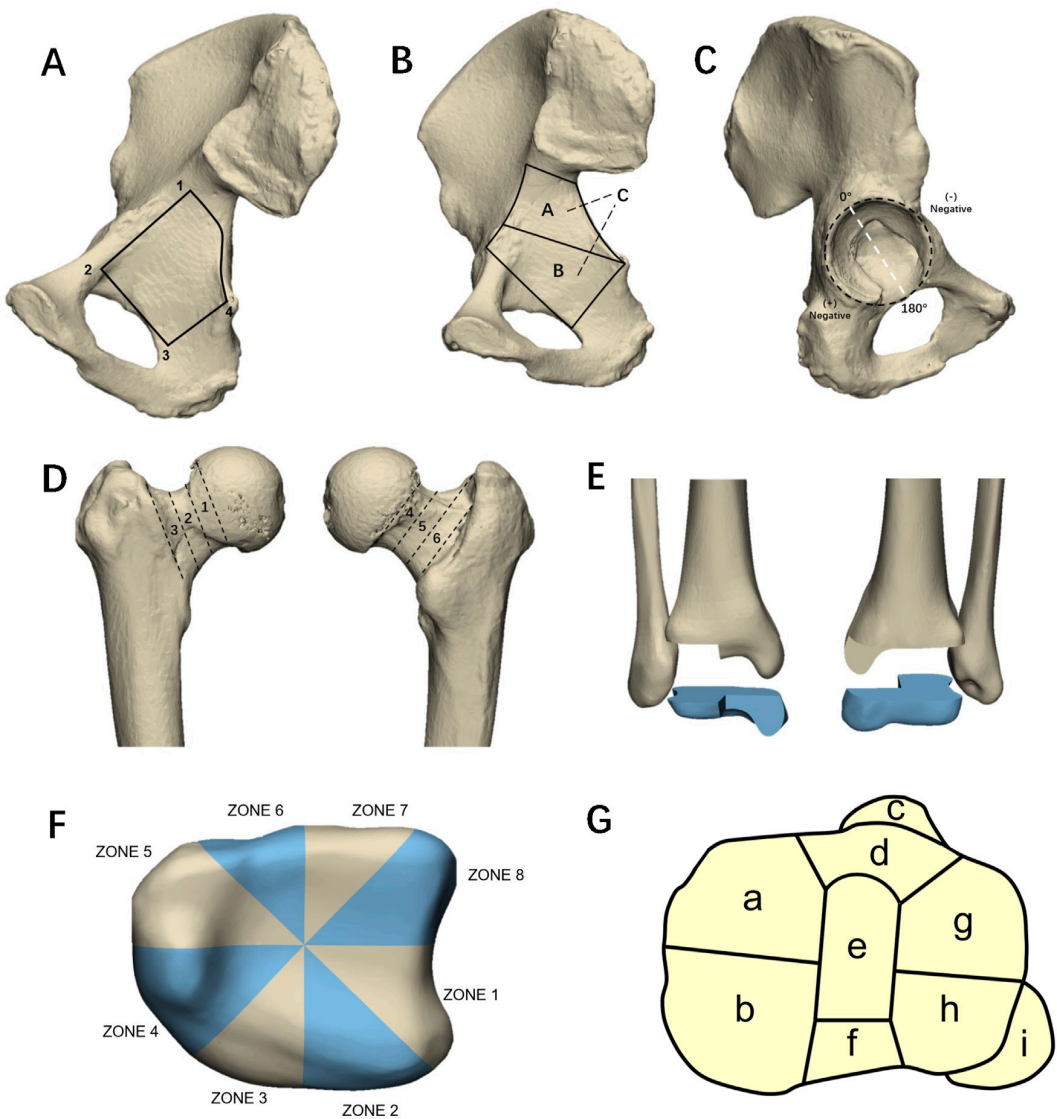
**(A)** The area enclosed by the four points (1–4) connected roughly represents the trapezoidal shape area of the quadrilateral board. **(B)** The schematic diagram of the new classification of tetrahedral fractures proposed by Yang et al. Zones A and B are bounded by a line from the ischial spine to the iliopubic eminence. **(C)** The clock face position is used to describe the location of fractures on the surface of the acetabulum joint. The midpoint of the transverse acetabular ligament serves as the +180° reference point, with the 0° reference point set perpendicular to the ligament. **(D)** Six-zone classification of femoral neck fractures. The anterior zones were categorized as subcapital (Zone 1), transcervical (Zone 2), and basocervical (Zone 3). Likewise, the posterior zones were identified as subcapital (Zone 4), transcervical (Zone 5), and basocervical (Zone 6). **(E)** Schematic diagram of a triplane fracture. **(F)** Labronici et al. divided the distal tibia into eight zones. **(G)** Tibial plateau nine-column fracture classification.

The position of the quadrilateral plate is shown in Figure 8A. Yang et al. (2018) first conducted a fracture mapping study of quadrilateral plate fractures, analyzing 238 cases and producing a 2D fracture map. The quadrilateral plate was divided into two sections, posterior “A” and anterior “B” (Figure 8B), demarcated by a line from the ischial spine to the iliopubic eminence. They discovered that 65% of the fractures intersected both sections. The fractures were further classified into three categories: those crossing the upper border of both zones (115 cases, 48%), those approximately perpendicular to the inner portion of the arch (110 cases, 46%), and those extending from the upper segment to the arch (60 cases, 25%). In 2019, Yang et al. (2019b) further analyzed the morphological characteristics in quadrilateral region for different acetabular fractures. Their research revealed that the fracture lines in double-column fractures predominantly occurred at the upper and posterior regions of the quadrilateral plate. Conversely, the fracture lines associated with transverse and posterior wall fractures were primarily located in the posterior region. T-shaped acetabular fractures (T-SAF) displayed a relatively uniform distribution of fracture lines across the region. In posterior column fractures, the fracture lines were primarily situated in the middle region, whereas in anterior column fractures, they were predominantly found in the upper region. Ye et al. (2022a) further investigated the morphology of double-column acetabular fractures within the quadrilateral region, corroborating the findings of Yang et al. Their study indicated that the majority of fractures in the quadrilateral region of double-column acetabular fractures were simple and typically located on the peripheral edges of the quadrilateral plate (Ye K. et al., 2022). The acetabular notch emerged as the most frequently affected area, followed by the posterior superior region, while the central region was seldom impacted (Ye K. et al., 2022). These studies highlight that different types of acetabular fractures exhibit distinct fracture line patterns, which are crucial for accurate diagnosis and effective treatment planning.

#### 4.1.2 Acetabular both-column fractures

Acetabular double-column fracture is usually caused by high energy injury, which is the second most common type of acetabular fracture, accounting for 20% of all acetabular fractures (Giannoudis et al., 2005; Ferguson et al., 2010). Due to the destruction of the normal anatomical structure of the acetabular roof and the loss of reference for fracture reduction and fixation, it is one of the most complex types of acetabular fractures (Yang et al., 2020). Yang et al. (2019a) revealed that the fracture lines in acetabular double-column fractures exhibit a Y-shaped distribution, consisting of three branches: one branch from a point near the anterior superior spine to the ischial spine, followed by a branch from the iliac crest to the acetabular roof, and a branche traversing the posterior wall. Unfortunately, they did not create a map of the acetabular articular surface. The condition of the acetabular articular surface is crucial for reduction purposes. Ye et al. (2023a) retrospectively analyzed 100 patients with double-column acetabular fractures and created 3D fracture maps of these fractures. They found that the fracture lines in double-column acetabular fractures exhibit a “dumbbell-shaped” distribution on the acetabular articular surface. These fracture lines start from the triangular region in the lower anterior aspect, where the superior pubic ramus and acetabular wall intersect, then extend through the junction between fossa and anterior lunate surface, and continue into the posterior region of the acetabulum (Ye et al., 2023a). Contrary to the findings of Ye et al., Yin et al. discovered that the fracture lines in double-column fractures are concentrated in the anterior inferior region of the acetabular articular surface, rather than the posterior superior region. Additionally, Yin et al. observed that the fracture lines outside the acetabular articular surface primarily extended from the iliac crest to the apex of the acetabulum, which is similar to the findings of Yang et al.

#### 4.1.3 Acetabular posterior wall fracture

Posterior wall fracture is the most common type of acetabular fracture, accounting for approximately 1/4 to 1/3 of all acetabular fractures (Zhang et al., 2014; Patel and Moed, 2017; Ahmed et al., 2018). When the hip joint flexes >60° and withstands sufficient violence, it is easy to cause posterior wall fractures. Zhao et al. (Cho et al., 2021a) conducted an analysis on the fracture morphology of acetabular posterior wall fractures using quantitative measurements of fracture characteristics. The study employed the midpoint of the transverse acetabular ligament as the +180° reference point, with the 0° reference point established perpendicular to the ligament, and designated the posterior acetabular surface as the positive area (Figure 8C). They defined the fracture span of the posterior acetabular surface as the ratio of the vertical distance between the fracture beak and acetabular rim to the total length from edge to rim. They found that 80.6% of fracture lines were distributed between 18.7°–117°. In addition, 61.7% of the fracture lines had a fracture span between 0.6 and 0.9. Tian et al. (2022) based on the injury mechanism of posterior wall fractures. They divided all patients into two groups: group A (both-column fractures and posterior wall fractures) and group B (including posterior column and posterior wall fractures; T-shaped fractures and posterior wall fractures; and transverse fractures and posterior wall fractures) (Tian et al., 2022). They found significant differences in fracture morphology between the two groups: the spatial displacement (farthest 3D distance of the fracture) in group A was smaller than that in group B, the fracture area of the articular surface in group A was larger than that in group B, and the fracture line in group B was smaller than that in group B. Group A is more upwardly distributed. Ye et al. (2023b) analyzed the morphology of the posterior elements of the acetabular both-column fracture. They divided the posterior edge of the posterior column and the corresponding posterior acetabular surface (RAS) into three equal parts (the cephalic, medial, and caudal), and analyzed the distribution of fracture lines on the RAS (Ye et al., 2023b). They found that the extra-articular fracture lines of posterior wall fractures were widely distributed throughout the posterior RAS and the cephalic ilium. The intra-articular fracture line originated from the superior rim of the acetabulum, passed downward through the junction of the lunate surface and the fossa, and finally ended in the lower posterior part of the joint. The area of the fracture line they reported was more extensive than that reported by Cho et al. for isolated posterior wall fractures. Their study found that more than two-thirds of transverse line fractures were located in the cephalic third of the RAS, providing the possibility of fracture reduction using an anterior approach (Ye et al., 2023b).

#### 4.1.4 Others fractures

Oguzkaya et al. (2023b) created a 2D fracture map encompassing all types of acetabular fractures. They classified these fractures into three categories: anterior, posterior, and complex fractures. The study revealed that fracture lines in anterior fractures were primarily horizontal and diagonal, with a higher concentration in the lower anterior region. In contrast, posterior fractures typically exhibited oblique lines situated between the acetabular dome and the mid-portion of the posterior wall. For complex fractures, the fracture lines were predominantly located just above the acetabular fossa, the acetabular dome, and the posterosuperior portion of the acetabulum (Oguzkaya et al., 2023b).

T-SAFs represent a complex category of acetabular fractures, constituting approximately 6% of all such injuries (Scheinfeld et al., 2015). T-SAF is characterized by two principal fracture lines: a transverse line that traverses the acetabulum, detaching it from the ilium, and a vertical line that intersects the transverse line below, extending through the anterior and posterior columns (Letournel, 1980). The configuration of these fracture lines resembles the letter “T,” which accounts for the designation of this fracture type. Ye et al. (2022b) developed a fracture map based on an analysis of 56 cases of T-SAF. Their findings indicated that the transverse fracture line typically meanders without compromising the acetabular roof. In contrast, the vertical fracture line tends to incline either anteriorly or posteriorly along the rim of the acetabular fossa, leading to the separation of the obturator foramen (Ye P. et al., 2022). Furthermore, their research revealed that the transitional zone of bone thickness is the most prevalent site for fractures in this context.

Pure transverse acetabular fractures are classified as a fundamental type in the Judet-Letournel system, characterized by their straightforward geometric fracture patterns. Li et al. (2022a) developed fracture map and heat map for 49 cases of transverse fractures, revealing that the anterior edges of these fractures tend to cluster narrowly, whereas the posterior lines are more widely dispersed (Li J. et al., 2022). Their study also highlighted that the distribution of fracture lines within the joint predominantly occupies the upper and middle thirds of the articular surface, with recurrent fracture lines affecting the weight-bearing dome primarily located in the posterior roof region.

### 4.2 The femur fractures

#### 4.2.1 Intertrochanteric fractures (IFs)

IFs are are a prevalent type of injury among the elderly, with intertrochanteric fractures accounting for up to 50% of these cases (Michelson et al., 1995; Socci et al., 2017; Shi et al., 2023). Because their injuries can result in high morbidity, disability, and mortality, intertrochanteric fractures are currently still considered an “unresolved fracture” type (Haidukewych, 2009; 2010; Valizadeh et al., 2012). Details of the studies on Malleolus fractures are summarized in Table 2.

**TABLE 2:** Fracture map study of the femur fractures.

Study	Fractures	Participant Characteristics	2D/3D	Software	Reduction of fractures	Reconstruction of fracture	Heat map
Fu et al. (2019)	Intertrochanteric fracture	Gender:42 male, 58 female Age:overall mean, 61y	2D	None	Yes	Yes	Yes
Ren et al. (2023b)	Intertrochanteric fracture	Gender144 male, 204 female Age:overall mean, 73.5y	3D	Mimics; 3-matic	Yes	Yes	No
Li et al. (2020b)	Intertrochanteric fracture	Gender37 male, 78 female Age:overall mean, 78 ± 7.98y	3D	Mimics; 3-matic	Yes	Yes	No
Li et al. (2019)	Intertrochanteric fracture	Gender105 male, 309 female Age:overall mean,80.2 ± 13.0y	2D	Mimics; Photoshop	Yes	Yes	No
Öğümsöğütlü and Kılınçoğlu (2023)	Femoral neck fractures	Gender59 male, 61 female Age:overall mean,67.92 ± 17.44y	2D	Essential Skeleton 4; Adobe Illustrator	Yes	Yes	No
Wu et al. (2022)	Femoral head fractures	Gender169 male, 40 female overall mean,39.85 ± 16.41y	3D	Mimics; 3-matic	Yes	Yes	Yes
Ding et al. (2022)		Gender33 male, 26 female None	3D	Mimics; 3-matic; E−3D	Yes	Yes	Yes
Li et al. (2022b)	AO/OTA types 33 distal femoral fractures	Gender92 male, 124 female Age: male,51.1 ± 13.7y female,66.4 ± 12.6y	3D	Mimics; 3-matic; E−3D	Yes	Yes	Yes
Chen et al. (2023)	AO/OTA 33A and 33C distal femoral fractures	Gender40 male, 34 female Age:overall mean,58 (18–92) y	3D	Mimics; 3-matic; E−3D	Yes	Yes	Yes
Xie et al. (2017)	Hoffa fractures	Gender52 male, 24 female Age:overall mean,50.4 (18–80)y	3D	Mimics; 3-matic	Yes	Yes	Yes

Fu et al. (2019) employed a 2D parameterized fracture probability heatmap to visualize and analyze 100 cases of IFs. They founded that the intertrochanteric line and intertrochanteric ridge were dense regions for fracture. Additionally, they observed an increased probability of greater trochanter fractures with advancing age (Fu et al., 2019). Osteoporosis increased the probability of IFs, although the fracture distribution was dispersed. Females exhibited greater bone loss, resulting in a wider distribution of IFs. The study by Li et al. (2019) also reached a similar conclusion. They found that most of the fracture lines of intertrochanteric fractures were located at the intertrochanteric line where the iliofemoral ligament is attached, the intertrochanteric ridge, and the greater trochanter where the gluteus medius muscle is attached. Similarly, the study by Li et al. (Li C. et al., 2020; Ren et al., 2023b) showed that the fracture lines were mainly concentrated in the lesser trochanter, greater trochanter, intertrochanteric line and intertrochanteric ridge area.

#### 4.2.2 Femoral neck fracture (FNFs)

FNFs are most common in elderly patients and are often caused by low-energy injuries such as falls (Johnell and Kanis, 2006). Femoral neck fractures in young patients are mostly caused by high-energy violent injuries, accounting for only 3% of patients with FNFs (Robinson et al., 1995). With the advancement of imaging technology and equipment, internal fixation materials and designs, treatment concepts and surgical techniques, the treatment effect of f FNFs has been significantly improved (Miller et al., 2014). However, the incidence of complications of femoral neck fractures, especially nonunion and avascular necrosis of the femoral head, remains high (Ly and Swiontkowski, 2008).

Öğümsöğütlü and Kılınçoğlu (2023) analyzed the morphology of 120 cases of FNFs. They divided the anterior and posterior regions of the femoral neck into three zones each (Figure 8D). They found that fractures most commonly affected Zone 4, while Zone 6 was the least susceptible. Zones 2 and 5 had significantly higher involvement in patients under the age of 65 (Öğümsöğütlü and Kılınçoğlu, 2023).

#### 4.2.3 Femoral head fractures (FHFs)

FHFs are rare hip injuries that predominantly occur in young adults and are typically caused by high-energy trauma (Chiron and Reina, 2022). These injuries can occur in isolation or as part of more complex hip injuries (Bernstein et al., 2022). Wu et al. (2022) reported the morphology characteristics of FHFs in 2022. They found that fractures were concentrated in the anterior inferior part of the femoral head surface, and fractures with anterior dislocation were mainly distributed on the upper lateral surface of the femoral head surface (Wu et al., 2022).

#### 4.2.4 Distal femoral fractures (DFFs)

DFFs are complex, accounting for 0.5% of all fractures and 6% of femoral fractures (Martinet et al., 2000; Elsoe et al., 2018). patients showed bimodal distribution, with high-energy trauma in young patients and low-energy trauma in elderly patients with osteoporosis (Elsoe et al., 2018).

Both Li et al. (2022b) and Chen et al. (2023) studied the fracture morphology of DFFs and their subtypes, and they reached similar conclusions. The fracture lines of distal femoral fractures were mainly concentrated in the metaphysis, the intercondylar notch and around the patellofemoral joint (Li R. et al., 2022; Chen et al., 2023). Among each subtype, the intercondylar fracture morphology of AO/OTA type B and C fractures was similar, whereas the supracondylar fracture pattern is different in AO/OTA types A and C. The fracture line of AO/OTA type A fracture is mainly located near the metaphysis, especially on the anterior surface of the supracondylar area (Li R. et al., 2022; Chen et al., 2023). In type B fractures, the fracture line was mainly concentrated around the intercondylar notch. The fracture line of type C fracture is mainly concentrated on the lateral side of the intercondylar notch and the metaphysis, with a “Y” shape distribution (Li R. et al., 2022; Chen et al., 2023).

Hoffa fracture is a coronal fracture posterior to one or both condyles of the distal femur, accounting for 8.7%–13% of distal femur fractures and is classified as AO/OTA 33-B3-2 or 33-B3-3 (Nork et al., 2005; Peez et al., 2024) This fracture is of great interest because it can be easily overlooked on routine imaging studies (Nork et al., 2005). Xie et al. (2017) determined the distribution location and frequency of Hoffa fractures through 2-D and 3-D CT technology in 2017. They found that Hoffa fractures most commonly occur in the middle third of the lateral condyle, extending from anteroinferior to posterosuperior and from anterolateral to posteromedial Ding et al. also observed such phenomena (Xie et al., 2017). Furthermore, Ding et al. (Ding et al., 2022) found that the lateral condyle α angle (the angle between the coronal fracture and the posterior condylar axis in the axial plane) and β angle (the angle between the coronal fracture and the femoral posterior cortex in the sagittal plane) in type 3-C3 fractures were significantly smaller than those in Hoffa fractures (B3), while there was no significant difference in the angle of the medial condyle. Additionally, the coronal fracture and articular comminution zone in type 33-C3 were more anteriorly located (Ding et al., 2022).

### 4.3 Malleolus fractures

Malleolar fractures represent approximately 3.9% of all fractures and are the most prevalent type of intra-articular fractures (Koval et al., 2005). The intricate anatomy of the ankle joint, coupled with diverse injury mechanisms such as vertical compression, horizontal torsion, and other indirect forces, results in a variety of fracture presentations. Zeng et al. (2022) conducted a retrospective analysis of 228 patients with malleolar fractures, employing 3D mapping. Their findings indicated that the concentration of fibular fracture lines predominantly occurred above the tibiofibular ligament, extending obliquely across the distal fibula from the anterior margin of the fibular neck. Additionally, the focal area of tibial fractures was primarily within the anterior tibial fornix, at the anterolateral corner, and the fibular notch (Zeng et al., 2022). Details of the studies on malleolus fractures are summarized in Table 3.

**TABLE 3:** Fracture map study of malleolar fractures.

Study	Fractures	Participant Characteristics	2D/3D	Software	Reduction of fractures	Reconstruction of fracture	Heat map
Quan et al. (2021)	Posterior malleolus fracture	Gender: male, 67 female Age:None	3D	Mimics; 3-matic; E−3D	Yes	Yes	Yes
Yu et al. (2021)	Malleolus fracture of supination-external rotation	Gender:26 male, 44 female Age:overall mean, 53.16 ± 15.36y	3D	Mimics; Photoshop	Yes	Yes	Yes
(Matthew et al., 2019)	Posterior malleolar fracture	Total number of participants:122	2D	Fireworks	No	No	No
Wang and Chang (2023)	Danis–Weber type B lateral malleolar fractures	Gender:46 male, 68 female Age: male, 54.0 ± 14.5y female,62.5 ± 12.9y	3D	Mimics; 3-matic	Yes	Yes	No
Su et al. (2020)	Posterior malleolar fractures	Gender:49 male, 63 female Age:overall mean, 49.0 ± 15.9y	3D	Mimics; 3-matic	Yes	Yes	No
Cao et al. (2022)	Lateral malleolus fractures	Gender:67 male, 81 female Age: unstable group,44,2 ± 17.7y stable group,52,7 ± 14.6y	2D	PACS/RIS MATLAB	No	No	No
Zeng et al. (2022)	Malleolar fractures	Gender:127 male, 101 female Age:overall mean, 42.6 (16–87)y	3D	Mimics 3-matic; Unigraphics; AutoCAD	Yes	Yes	Yes
Hadad et al. (2018)	Triplane fractures	Gender:20 boy,13 girl Age: boy,14.5 ± 0.7y girl,12.2 ± 0.9y	2D	3D Slicer; Photoshop; MATLAB	No	No	Yes

#### 4.3.1 Posterior malleolus fracture

Previous studies on malleolus fractures have predominantly focused on a subset of these injuries. Among these, fractures of the posterior malleolus are the most prevalent, accounting for approximately 7%–44% of all ankle injuries. These fractures frequently occur in conjunction with injuries to the medial and lateral malleoli or ligament damage, presenting a more severe clinical scenario than malleolus fractures that do not involve the posterior malleolus (Koval et al., 2005; Warner et al., 2014). Regarding the morphology of posterior malleolus fractures, there is a general consensus in the literature. The fracture line typically extends obliquely across the posterolateral articular surface of the distal tibia (Mitchell et al., 2019; Su et al., 2020; Quan et al., 2021; Yu et al., 2021). Mitchell et al. (Mitchell et al., 2019) examined the morphology of posterior malleolus fractures and determined that these fractures, when associated with spiral fractures of the distal tibia, consistently exhibited a posterolateral oblique orientation. Su et al. (2020) observed that medial malleolus fractures were present in all cases of posterior malleolar fractures. Furthermore, they compared supination-external rotation ankle fractures with pronation-external rotation ankle fractures and found that the latter displayed larger posterior tibial fragments. Additionally, there was a significant difference in the distribution of the lateral malleolar fracture line between the two groups; specifically, the lateral malleolar fracture line in pronation-external rotation ankle fractures was positioned higher (Su et al., 2020). Quan et al. (Quan et al., 2021) found that most posterior malleolus fractures are single-fragment fractures, consisting of two different sizes of fragments. The larger fragment always originates from the groove of the posterior tibialis posterior and flexor digitorum longus, and terminating at the middle part of the fibular notch. The smaller fragment mainly originates from one-half of the f posterior malleolus fracture, involving one-fourth of the fibular notch (Quan et al., 2021). Yu et al. (Yu et al., 2021) reported the distribution of posterior malleolar fracture line of supination-external rotation ankle fracture. They found that the average ratio of posterior fracture fragment area to total articular surface area was 14.96%, and most of the fracture lines were posterolateral oblique.

#### 4.3.2 Triplane fractures

Triplane fractures refer to distal tibial fractures that involve coronal, sagittal, and axial plane fractures of the epiphysis (Figure 8E). (Schnetzler and Hoernschemeyer, 2007) This fracture is specific to adolescents and is often referred to as transitional injury (Wuerz and Gurd, 2013). It is relatively uncommon, accounting for 5%–10% of ankle injuries in adolescents, with slightly higher incidence in boys than girls (Wuerz and Gurd, 2013). In 2018, Hadad et al. (2018) reported on the morphological characteristics of 33 cases of triplane fractures in children. Their findings revealed that metaphyseal fractures typically presented as medial-lateral fracture lines in the posterior metaphysis. Additionally, the epiphyseal fractures predominantly displayed a dense area in an inverted “three-pointed star” configuration. The study further indicated consistent patterns in the trajectory of the fracture lines among the patients. In all 33 cases, at least one fracture line exited through the anterior metaphysis. Specifically, the fracture lines exited through the medial malleolus in 11 cases, entered the posteromedial metaphysis in 24 cases, and exited through the posterolateral metaphysis in 19 cases (Hadad et al., 2018).

#### 4.3.3 Lateral malleolus fractures

Cao et al. (2022) developed 3D fracture map of lateral malleolus fractures to predict ligamentous injuries in supination external rotational malleolus fractures. They divided 148 patients into two groups: an unstable group comprising 41 cases, and a stable group consisting of 107 cases. Their analysis revealed that fracture lines in both groups descended from a posterior-upper to an anterior-lower direction. However, the fracture lines in the unstable group were significantly higher than those in the stable group. Furthermore, they identified fracture height and fracture inclination as critical predictive factors for syndesmotic instability, indicating that increased fracture height and inclination correlate with a greater risk of syndesmotic instability (Cao et al., 2022).

#### 4.3.4 Other fractures

Wang and Chang (2023) described the morphological characteristics of Danis-Weber type B lateral malleolus fracture. They found that the apex of nearly half of type B fractures were not on the posterolateral surface, which could damage the mechanical application of antiglide plates (Wang and Chang, 2023). The steeper fracture line and longer fracture spike indicate that the posterior-medial distribution of the fracture tip was further posterior, indicating a greater severity of violence and accompanying injuries. Additionally, they observed that these morphological features—steeper fracture lines and elongated fracture apices—correlated with a more posterior positioning of the sharp fracture end on the posterior-medial side, further implying greater violence and more severe accompanying injuries (Wang and Chang, 2023).

### 4.4 Tibial plateau fractures (TPFs)

TPFs account for 1% of all fractures and are considered to be one of the most challenging intra-articular fractures (Lowe et al., 2011; Elsoe et al., 2015). It is a complex injury caused by high-energy trauma (Wasserstein et al., 2014). The Schatzker classification based on X-ray is the most commonly used classification system for TPFs. Details of the studies on Malleolus fractures are summarized in Table 4.

**TABLE 4:** Fracture map study of tibial plateau fractures (TPFs).

Study	Fractures	Participant Characteristics	2D/3D	Software	Reduction of fractures	Reconstruction of fracture	Heat map
Yao et al. (2022a)	TPFs	Gender:204 male, 142 female Age: male,44.8y; female 50.8y	3D	Mimics; 3-matic	Yes	Yes	No
Yao et al. (2022b)	Hype-rextension TPFs	Gender:663 male, 580 female Age: overall mean, 52.2 ± 13.1y	3D	Mimics; 3-matic; E−3D	Yes	Yes	Yes
Kerschbaum et al. (2021)	TPFs	Gender:170 male, 108 female Age: male,46.3y; female 53.5y	2D	PowerPoint	No	No	No
Molenaars et al. (2015)	TPFs	Gender:170 male, 108 female Age: male,46.3y; female 53.5y	2D	Firework	No	No	No
Molenaars et al. (2019)	TPFs	Gender:17 male, 30 female Age: overall mean,49 (21–89) y	2D	Rhinoceros	No	No	No
Yao et al. (2020)	TPFs	Gender:411 male, 348 female Age: male,49.7 ± 12.3 y female, 53.7 ± 13.4 y	3D	Mimics; 3-matic; E−3D	Yes	Yes	Yes
Kabra et al. (2023)	TPFs	Gender:206 male,21 female Age: overall mean,41.5 ± 9.38y	3D	Mimics; 3-matic	Yes	Yes	No
Chen et al. (2016)	TPFs	Gender:106 male,80 female Age: overall mean, 54.9 (17–87) y	2D	Fireworks	No	No	Yes
McGonagle et al. (2019)	TPFs	Gender:80 male,41 female Age: overall mean, 45.5 (21–77) y	2D	None	No	No	No

#### 4.4.1 Studies including all types of TPFs

Most studies on morphology of TPFs have come to a consistent conclusion: the fracture lines are mainly concentrated around the lateral condyle and tibial spine. Kerschbaum et al. (2021) created 2D maps of 271 patients with TPFs. They found that the fracture lines of TPFs were mainly distributed in the lateral column and central column of the tibial plateau. A 3D fracture map study by Yao et al. (2020) reached a similar conclusion, showing that the fracture lines were mainly concentrated at the insertion of the anterior and posterior cruciate ligament and the medial side of the lateral condyle. In addition, Yao et al. proposed a nine-column classification for TPFs based on fracture heatmaps. They think that this classification could encompass all fracture types of the lateral and medial columns. Another 3D fracture map study by Yao et al. (Yao P. et al., 2022) reached a similar conclusion: the dense fracture line was mainly located at the medial edge of the lateral condyle. In addition, the fracture line forms a high-density accumulation area between the intercondylar eminence and the anterior edge of the fibular head. Furthermore, they discovered that different types of TPFs exhibited distinct distribution characteristics of fracture lines. Kabra et al. (2023) conducted a retrospective analysis of imaging data from 228 cases of proximal tibia fractures and observed that the fracture lines were predominantly situated around the tibial tubercle, in the sagittal plane above the lateral plateau, and in the coronal plane above the medial plateau (Kabra et al., 2023).

In addition, several studies have conducted comparative analyses of the fracture morphology among different types of TPFs. Molenaars et al. (Molenaars et al., 2015) created a 2D fracture map for tibial plateau fractures and analyzed the fracture patterns of lateral (Schatzker types I, II, and III), medial (Schatzker types IV), and bicondylar fractures (Schatzker types V and VI) (Molenaars et al., 2015). They observed that most fracture lines of lateral condyle fractures are located on the lateral edge of the lateral condyle, many fracture lines of medial condyle fractures leave from the posterior aspect of the lateral side of the plateau, and fracture lines of bilateral condyle fractures are mainly located on tibial spines and the outer margin of the lateral condyle (Molenaars et al., 2015). Chen et al. (Chen et al., 2016) conducted a retrospective analysis of CT data from 186 cases of tibial plateau fractures. Differing from previous studies, they delineated the most common depression areas and measured the depth of the depressions. They found that the depression area for Schatzker type III fractures was located in the anterolateral tibial plateau, Schatzker type V fracture was most likely to cause posterolateral depression, while Schatzker type VI fracture occurred in the center of the tibial plateau. They also found that there were significant differences in depression depth among different types of fractures, and the order of average depression depth was Schatzker type III, II, V, VI and IV (Chen et al., 2016).

McGonagle et al. (2019) generated 2D fracture maps for TPFs. However, they simplified the fracture lines as straight lines based on the entry and exit coordinates of the fracture lines. In reality, many fracture lines are curved or have an “S” shape, so their fracture maps may not accurately depict the actual fracture morphology.

#### 4.4.2 Hyperextension TPFs

Yao et al. (2022b) conducted a study in which they generated fracture maps and heatmaps specifically for hyperextension TPFs. Their findings indicated that fracture lines predominantly occurred at the anterior edge of the tibial plateau, with minimal involvement of the posterior articular surface. The primary characteristics of hyperextension tibial plateau fractures included anterior compression and posterior avulsion injuries.

#### 4.4.3 Other fractures

Molenaars et al. (2019) studied the articular coronal fracture angle of posterior medial tibial plateau fragments and found that the average angle for posterior medial articular fractures was 44°, with an average articular surface area encompassing 34% of the entire tibial plateau. There were no significant differences in the morphology of posterior medial fragments among Schatzker type IV, V, and VI fractures (Molenaars et al., 2019).

### 4.5 Humeral fractures

#### 4.5.1 Proximal humerus fractures (PHFs)

PHFs account for 5% of all fractures and are more commonly in elderly women with osteoporosis (Bergdahl et al., 2020). Most research on fracture mapping in proximal humerus fractures (PHFs) has been confined to a particular fracture type. Details of the studies on Malleolus fractures are summarized in Table 5. In 2019, Hasan et al. (Hasan et al., 2017) retrospectively collected 3D CT data from 48 cases of complex PHFs, transcribing these fractures onto a proximal humeral template to generate 2D fracture maps. Their findings indicated a high incidence of intra-articular fractures, with 52% involving the articular surface, predominantly located at the greater tuberosity, especially at the junction of the supraspinatus and infraspinatus tendon insertion sites. In contrast, fractures infrequently affected the bicipital groove. However, Misir et al. (2023) observed that all OTA/AO 11C3 fractures of the PHFs involved the bicipital groove, a discrepancy that may be due to variations in average age, gender, and prevalence of osteoporotic fractures among the studied populations. Hasan et al. (2017) were the first to elucidate the relationship between PHF morphology and local bone markers alongside soft tissue attachments. Similarly, Mys et al. (2023) discovered that fracture lines predominantly occurred in the surgical neck and between tuberosities and tendon insertions, with rare involvement of the bicipital groove and rotator cuff tendon insertions. They also noted that fractures of the articular surface were uncommon, contrasting with findings from Hasan et al. and Misir et al., Ren et al. (Ren et al., 2023a) classified PHFs into six groups and created fracture maps for each type, but did not further analyze the morphology and distribution of the fracture lines.

**TABLE 5:** Fracture map study of humeral fractures.

Study	Fractures	Participant Characteristics	2D/3D	Software	Reduction of fractures	Reconstruction of fracture	Heat map
Mys et al. (2023)	Complex PHFs	Gender:40 male, 10 female Age:overall mean, 68.5 ± 13.1 y	3D	Matlab; Amira 3D; Procrustes	Yes	Yes	Yes
Ju et al. (2023)	Three- and four-part PHFs	Gender:12 male, 59 female Age:overall mean,69.0 ± 13.0y	3D	Mimics; 3-matic	Yes	Yes	No
Ren et al. (2023a)	PHFs	Gender:121 male, 191 female Age:overall mean,67.3y	3D	Mimics; 3-matic	Yes	Yes	No
Guo et al. (2023)	Bony Bicipital Groove Fractures	Gender:121 male, 191 female Age:overall mean,67.3y	3D	Mimics; Adobe Illustrator	No	Yes	Yes
Hasan et al. (2017)	Complex PHFs	Gender:12 male, 36 female Age:overall mean,62 (21–88) y	2D	Essential Skeleton; Adobe illustrator; OsiriX	No	Yes	No
Misir et al. (2023)	Complex PHFs	Gender:95 male, 106 female Age:overall mean, 57.5 ± 17.7y	2D	3D Slicer; Photoshop CC; Adobe Illustrator	No	No	Yes
Wang et al. (2021)	Distal humerus fracture	Gender:59 male, 43 female Age: male,37.1 ± 16.7y emale,56.2 ± 15.0y	3D	Mimics; 3-matic; E−3D	Yes	Yes	Yes

Furthermore, several scholars have reported on the fracture morphology of specific locations in PHFs. Ju et al. (2023) reported on the fracture morphology of greater tuberosity fractures in three-part and four-part PHFs. They found that the fracture morphology of the large tubercle was similar between the three-part and the four-part PHFs. The fracture line had three branches in the greater tubercle, which were located in the front, middle and back of the great tubercle. The fracture lines within the greater tuberosity exhibit three branches, located anteriorly, medially, and posteriorly, resulting in an overall fan-shaped distribution. Guo et al. (2023) classified the bicipital groove (BG) into three sections: the upper 1/3, middle 1/3, and lower 1/3, and analyzed the fracture morphology of the bicipital groove in proximal humerus fractures. They found that bicipital groove fractures were predominantly located in the upper two-thirds of the BG, particularly in the middle third.

#### 4.5.2 Distal humeral fractures

Distal humeral fractures make up approximately 2% of elbow fractures in adults (Bégué, 2014). Wang et al. (Wang et al., 2021) were the pioneers in employing 3D mapping to elucidate the morphology of distal humeral fractures. Their research identified that the predominant fracture zones were located in the radial fossa, coronal fossa, olecranon fossa, and the external segment of the trochlea (Wang et al., 2021). These insights are crucial for guiding the selection of treatment plans and the design of surgical fixations.

### 4.6 Calcaneal fractures (CFs)

CFs account for approximately 1%–2% of all fractures (Giannini et al., 2016). Approximately 75% of CFs are intra-articular (Marouby et al., 2020).More than 60% of calcaneal fractures are caused by axial loads following a fall from a height (Zhang et al., 2023). Due to the complexity of the fracture pattern, there is a high rate of complications, including wound infection, plantar fasciitis, and post-traumatic arthritis (Chen et al., 2011; Kissel et al., 2011; Seat and Seat, 2020). Therefore, in-depth research on the mechanisms of calcaneal fractures remains important to improve treatment. Details of the studies on Malleolus fractures are summarized in Table 6.

**TABLE 6:** Fracture map study of calcaneal fractures.

Study	Fractures	Participant Characteristics	2D/3D	Software	Reduction of fractures	Reconstruction of fracture	Heat map
Yu et al. (2022)	Calcaneal fracture	Gender:153 male, 57 female overall mean, 47.6 (19–72) y	2D	Mimics Photoshop	No	Yes	Yes
Zhang et al. (2023)	Calcaneal fracture	Gender:69 male, 21 female Age: None	3D	Mimics; 3-matic; E−3D	Yes	Yes	Yes
Shi et al. (2022)	Calcaneal fracture	Total number of participants:67 Age:None	3D	Mimics; 3-matic	Yes	Yes	No
Guo et al. (2021)	Sanders Type 2 Joint Depression Calcaneal Fractures	Age: overall mean, 45.7y	3D	E−3D	Yes	Yes	Yes
Ni et al. (2021)	Complex intra-articular calcaneal fractures	Gender:47male, 15 female overall mean, 46.2 (20–58) y	3D	Mimics; 3-matic; E−3D	Yes	Yes	Yes
Shi et al. (2023)	Calcaneal fractures	Gender: 59male, 8 female Age: fmale,43.98 ± 10.87y female,48 ± 17.33y	3D	Mimics 3-matic; E−3D	Yes	Yes	Yes

In 2021, Ni et al. (2021) used fracture mapping to describe the fracture line distribution of CFs for the first time. They found that CFs mainly involve the anterior edge of the posterior joint and extend medially, posteriorly and anteriorly, and rarely involve the posterior tuberosity and anterior process of the calcaneus. In 2023, Zhang et al. (2023) drew fracture maps of 90 cases of intra-articular calcaneal fractures, and they obtained similar results to Ni et al. They found that the fracture lines were concentrated at the Gissane angle and posterior articular surface of the calcaneus. Similarly, Shi et al. (2022) found that the fracture line was mainly concentrated at the angle of Gissane and extended posteriorly to the posterior part of the lateral tuberosity and the anterior part of the medial process of the calcaneal tuberosity (Shi et al., 2022). In the axial 3D map view, the hot zone of the fracture line was mainly concentrated in the anterior area of the posterior articular surface. In addition, they also found that calcaneal fractures rarely involve the posterior calcaneal tuberosity and anterior process, which is consistent with the report by Ni et al.

Guo et al. (2021) analyzed Sanders type II joint depression fracture and tongue-type fracture respectively by fracture mapping. In addition to describing the overall situation of the calcaneal fracture line, they also described the fracture line of the calcaneal protrusion in detail, and put forward different fixation recommendations for different subtypes of fractures (Guo et al., 2021). In 2022, Yu et al. (2022) drew 2D fracture maps of six sections for intra-articular calcaneal fractures. This study was the first to link the distribution of calcaneal fracture lines with the internal structure of the calcaneus, indicating that the anatomy of the talus and calcaneus as well as the internal structure of the calcaneus play a crucial role in CFs.

Shi et al. (2023) used 2D and 3D fracture mapping to describe the frequency and displacement of sustentacular fractures in intra-articular CFs. Their research findings indicate that sustentacular fragments within CFs are not “constant’ fragments” (Shi et al., 2023). This finding refutes the theory that the sustentacular fragments are “constant.” (Shi et al., 2023).

### 4.7 The radius fractures

#### 4.7.1 Radial head fracture

Radial head fracture is a common elbow injury, with a common mechanism of injury being a fall with an outstretched arm (Duckworth et al., 2012b). Mellema et al. (2016) used quantitative 3D CT reconstruction to determine the fracture line distribution and location of 66 intra-articular radial head fractures. They found that fracture lines were concentrated in the anterolateral quadrant of the radial head, with less accumulation in the posteromedial quadrant. Not only that, they conducted a qualitative and quantitative analysis of the fracture lines and found that the distribution and location of fracture lines did not differ between specific fracture patterns in the elbow. Details of the studies on Malleolus fractures are summarized in Table7.

**TABLE 7:** Fracture map study of the radius fractures.

Study	Fractures	Participant Characteristics	2D/3D	Software	Reduction of fractures	Reconstruction of fracture	Heat map
Mellema et al. (2016)	Fractures of the radial head	Gender:38 male, 28 female Age; overall mean, 49 ± 15y	2D	3D Slicer; Rhinoceros Fireworks	Yes	Yes	No
Zhang et al. (2019)	Distal radius fractures	Gender:17 male, 23 female overall mean, 61.1 (25–85) y	2D	Mimics Photoshop	Yes	Yes	Yes
Misir et al. (2018)	Distal radius fractures	Gender:23 male, 11 female overall mean, 56 (39–72) y	2D	Adobe Illustrator	No	No	No
Li et al. (2020a)	Volar lunate facet fractures of the distal radius	Gender: 26 male, 33 female Age: overall mean, 50.1 (18–69) y	3D	Mimics 3-matic	Yes	Yes	Yes
Clarnette et al. (2022)	Comminuted distal radius fractures	Gender:11 male, 12 female overall mean, 51 ± 18y	3D	Mimics; 3-matic	Yes	Yes	Yes

#### 4.7.2 Distal radius fractures (DRFs)

DRFs are one of the most common fracture types in the emergency department, with intra-articular fracture accounting for approximately 25% of all DRF (Gordon et al., 2003). Misir et al. (2018) drew 2D fracture maps of 34 cases of OTA/AO 23C3 fractures. They found that the fracture lines were mainly distributed in the central area of the distal radius articular surface. However, Li et al. (Li S. et al., 2020) obtained different results from Misir et al. They also created fracture maps for AO/OTA type 23C3 distal radius fractures and found that the fracture lines formed a cross pattern on the articular surface, mainly concentrated in the posterior two-thirds of the joint surface and the dorsal ridge of the lunate fossa. Additionally, Li et al. (Li C. et al., 2020) think that the Melone classification system did not adequately represent all C3 fractures. This finding also reveals the shortcomings of the Melone typing system.

Zhang et al. (2019) created 2D fracture maps and heat maps of intra-articular fractures of the DRFs using simplified 3D CT reconstructions. They found that the highest intensity of fracture lines was distributed in the posterior aspect of the joint, forming an inverted “T” shape region. This finding is consistent with the results reported by Li et al., (Zhang et al., 2019), The keystone projected area, the radial styloid process and the metacarpal radial side articular surface were the least affected by fractures.

In addition, Clarnette et al. (2022) generated 2D fracture maps for 23 cases of volar Lunate facet fractures of the distal radius. They found that the fragments of volar lunate facet fractures were displaced towards the palmar and proximal directions, and the lunate bone remained connected to the displaced fragments (Clarnette et al., 2022). However, this study included only 23 patients, and the reliability of the results is uncertain.

### 4.8 Pilon fractures

Pilon fractures account for 10% of all lower extremity fractures (Aneja et al., 2018). Pillon fracture is defined as a fracture involving the articular surface of the distal tibia (Zelle et al., 2019; Bastias and Lagos, 2020). This is a complex fracture with severe soft tissue injury caused by axial high-energy trauma on the distal tibial plateau (Tomás-Hernández, 2016). At present, most of the mapping studies on Pilon fractures are 2D studies, and are limited to AO/OTA 43C Pilon fractures. The AO/OTA 43C Pilon fracture is the most complex type of Pilon fracture, caused by high-energy violence, and usually results in a comminuted intra-articular fracture (Marsh et al., 2007; Assal et al., 2015). Details of the studies on Malleolus fractures are summarized in Table 8.

**TABLE 8:** Fracture map study of Pilon fractures.

Study	Fractures	Participant Characteristics	2D/3D	Software	Reduction of fractures	Reconstruction of fracture	Heat map
Cole et al. (2013)	The OTA/AO Type 43C Pilon Fracture	Gender: 25 male, 13 female Age:overall mean, 42 (16–73) y	2D	Macromedia Fireworks	No	No	No
Gao et al. (2024)	The OTA/AO Type 43C Pilon Fracture	Gender: 91 male, 14 female Age: C1, 46.8 (23–71) y C2,50.5 (28–70) y C3,42.4 (15–71) y	3D	Mimics 3-matic Photoshop OpenCV	Yes	Yes	Yes
Labronici et al. (2021)	Pilon Fractures	Gender: 60 male, 13 female Age:overall mean, 43 (18–86) y	2D	None	No	No	No
Lim et al. (2023)	Pilon Fractures	Gender: 79 male, 17 female Age:overall mean,46.3 (27–66) y	3D	PowerPoint Photoshop	No	No	No

Cole et al. (2013) developed 2D fracture maps of 38 AO/OTA 43C3 Pilon fractures, identifying a consistent fracture pattern predominantly characterized by three major fragments: anterior, medial, and posterior. The fracture lines, which formed a “Y” shape, predominantly extended from the fibular notch to exit anteriorly and posteriorly near the medial malleolus. Similarly, Gao et al. (2024) conducted a 3D mapping study of 105 AO/OTA type 43C Pilon fractures, confirming the presence of major fracture lines extending from the fibular notch to the anterior or posterior borders of the medial malleolus (malleolus sulcus area).Unlike the qualitative approach of Cole et al., Gao et al. quantitatively analyzed the fracture lines and representative fragments, finding that the height of anterior and sagittal fragments in C2 Pilon fractures was greater than in C3 fractures (Gao et al., 2024). In a distinct study, Labronici et al. (2021) examined the impact of gender and varus or valgus displacement on Pilon fracture morphology. They divided the distal tibial articular surface into eight zones to assess the frequency of fracture lines in each (Figure 8F). Their findings indicated a higher severity of Pilon fractures in males compared to females, with most injuries in females affecting only two zones (69.2%), whereas only 26.7% of fractures in males were limited to two zones (Labronici et al., 2021). Furthermore, they observed distinct patterns associated with varus and valgus displacements; varus fractures typically exhibited a “Y” shape, whereas valgus displacement fractures appeared more random. Notably, the probability of a Pilon fracture affecting zone 1 (anterolateral region of the distal tibia) was 4.67 times higher in cases with valgus displacement than in those with varus displacement (*p* = 0.002) (Labronici et al., 2021). Lim et al. (2023) reported a correlation between fibular injury pattern and Pilon fracture morphology. They found that the dense area of fracture line was located in the anterolateral quadrant, and the pilon fractures in the fibula fracture group were more severe than those in the non-fractured fibula group. Lim et al. suggested that the injury pattern of the fibula can estimate the severity of Pilon fractures (Lim et al., 2023).

### 4.9 Patellar fractures

Current mapping studies on patellar fractures are limited to OTA/AO 34C type patellar fractures, and these studies have reached roughly the same conclusion (Table 9). The fracture lines of OTA/AO 34C type patellar fractures are mainly concentrated in the middle and lower areas of the patella. Simple fractures were distributed horizontally, and the fracture lines of complex fractures were randomly distributed (Misir et al., 2020; Cho et al., 2021b; Zhan et al., 2021; Wang et al., 2023). In each subtype, the distribution of fracture lines is slightly different. In type C1 and C2 fractures, the fracture lines at the medial and lateral articular surfaces are transverse or oblique. In contrast, for type C3 fractures, the medial articular surface is dominated by transverse and vertical fracture lines, while the lateral articular surface is dominated by transverse and oblique fracture lines (Misir et al., 2020).

**TABLE 9:** Fracture map study of the patellar fractures.

Study	Fractures	Participant Characteristics	2D/3D	Software	Reduction of fractures	Reconstruction of fracture	Heat map
Wang et al. (2023)	Patellar fractures	Gender:40 male, 48 female Age: overall mean, 57.2 (25–82) y	2D	E−3D; Photoshop	Yes	Yes	No
Cho et al. (2021b)	Patellar fractures	Gender:25 male, 13 female Age: overall mean, 51.7 (19–70) y	2D	Mimics; Photoshop	Yes	Yes	Yes
Zhan et al. (2021)	Patellar fractures	Gender:109 male, 78 female Age: overall mean, 53.18 (16–88) y	3D	Mimics; 3-matic; E−3D	Yes	Yes	Yes
Misir et al. (2020)	Patellar fractures	Gender:69 male, 14 female Age: overall mean, 52 (18–79) y	2D	3D Slicer; Photoshop	No	No	No

### 4.10 Scapula fractures

Scapula fractures are relatively rare, accounting for approximately <1% of all fractures (Schmidt et al., 2024). Scaphoid fractures are traditionally recognized as high-energy fractures and may serve as an indicator of severe comorbid injuries (Monica et al., 2016). Details of the studies on scapula fractures are summarized in Table 10. Armitage et al. (2009) first used 3D CT reconstruction in 2009 to study fracture morphology in 90 cases of scapula fractures. They found that the lateral margin below the glenoid process, the spinoglenoid notch, and the glenoid cavity (the medial side entering the scapular body) were dense zones of fracture (Armitage et al., 2009). The analysis results of Yimam et al. (2022) are consistent with those of Armitage et al. in varying degrees. However, the two studies differed slightly in terms of the frequency of exit zones at the lateral border. In Armitage et al., the most common fracture exit zones in the lateral border was second only to the glenoid, whereas Yimam et al. found that the lateral border was the most common exit zones. The second difference is that, according to Armitage et al., 17% of the fracture lines were on the surface of the glenoid joint, while in Yimam et al.'s study, the authors observed twice that percentage (34%). In another study, Cole et al. (2024) drew a 3D map of 87 scapular fractures in 2024, showing the morphology of scapular fractures more visually. According to their report, the primary fracture line run across the scapular body, starting from the superior lateral edge and exiting below the spinomedial angle. The results of the analysis are consistent with their previous studies and those of Armitage et al.,

**TABLE 10:** Fracture map study of the other fractures.

Study	Fractures	Participant Characteristics	2D/3D	Software	Reduction of fractures	Reconstruction of fracture	Heat map
Cole et al. (2024)	Scapular fractures	Gender:37 male, 78 female Age: overall mean, 78 ± 7.98y	3D	Mimics; Geomagic	Yes	Yes	No
Armitage et al. (2009)	Scapular fractures	Total number of participants:87 Age: overall mean, 78 ± 7.98y	2D	Firework	No	No	No
Yimam et al. (2022)	Scapular fractures	Gender:57 male, 11 female Age:overall mean, 40 (17–66) y	2D	Mimics; 3-matic; GIMP; Matplotlib	Yes	Yes	Yes
Oguzkaya et al. (2023a)	Sacral fractures	Gender:40 male, 32 female Age:overall mean, 46.5 ± 19.9y	2D	3D Slicer; Photoshop	No	No	Yes
Wang et al. (2024)	Talus fractures	Gender:83 male, 43 female Age:overall mean, 38.78y	3D	Mimics; 3-matic; E−3D	Yes	Yes	Yes
Chao et al. (2023)	Diaphyseal clavicular fractures	Gender:72 male, 28 female Age: male,55.8 ± 15.1y female,62.3 ± 12.1y	2D	3D Slicer	Yes	Yes	Yes
He et al. (2022)	The avulsion fractures of the fifth metatarsal base	Gender:125 male, 97 female overall mean, 49.1 ± 14.33y	3D	Mimics E−3D	Yes	Yes	Yes
Turow et al. (2020)	Scaphoid fractures	Gender:None Age:overall mean, 36 (18–84) y	2D	3D Slicer; Rhinoceros; Artec Studio	Yes	Yes	Yes
Mellema et al. (2014)	olecranon fractures	Gender:33 male, 45 female Age:overall mean, 62y	2D	3D Slicer; Rhinoceros; Fireworks	Yes	Yes	Yes

### 4.11 Ulna fractures

Olecranon fractures account for 10% of all upper extremity fractures and 18% of all proximal forearm fractures (Veillette and Steinmann, 2008; Duckworth et al., 2012a). Bart et al. (Lubberts et al., 2017) characterized the morphology of olecranon intra-articular fracture by mapping technique. They found that the fracture lines of non-displaced or slightly displaced fractures and posterior dislocation fractures were concentrated at the base of the coronoid and the trochlear notch, while those of displaced fractures and anterior fractures and dislocations were more widely distributed in the depths of the trochlear notch (Table 10).

Mellema et al. (2014) reconstructed 110 coronoid fractures models and produced 2D fracture maps and heat maps of coronoid fractures to determine whether the type of coronoid fractures was associated with a specific overall traumatic elbow instability injury pattern. According to their report, the fracture line was most commonly found in the volar half of the radial ulnar notch, and the association between coronoid fracture type and elbow fracture-dislocation pattern was significant (Mellema et al., 2014).

### 4.12 Sacral fractures

Oguzkaya et al. (2023a) analyzed the fracture morphology of three types of sacral fractures, and they found that in zone I fractures, most fracture lines were oriented vertically and obliquely (up to 45°) on both sides. In zone II fractures, the fracture lines are concentrated at the S1 and S2 levels. The anterolateral and posterolateral portions of the sacrum are less affected in right-sided fractures. In zone III fractures, the fractures were concentrated around the sacral canal at the S1, S2, and S3 levels. The median sacral crest and midline were largely unaffected (Oguzkaya et al., 2023a).

### 4.13 The fractures of the fifth metatarsal base

He et al. (2022) reported the fracture morphology of the fractures of the fifth metatarsal base and proposed a new classification. The authors found that the fracture lines for the avulsion fractures of the fifth metatarsal base were mainly concentrated in the dorsal arc zone. They developed a classification based on fracture patterns and soft tissue insertion, which helps to guide the mechanism of soft tissue involvement and potential injury: Type I is mainly concerned with the role of the lateral zone of the plantar fascia; type II is mainly related to the role of the short peroneus brevis; type IIIA is related to the combined action of the peroneus brevis and the lateral fascia bundle of the plantar fascia, with one fracture line, and type IIIB involves the combined action of the peroneus brevis and the lateral fascia bundle of the plantar fascia, with two fracture lines (He et al., 2022).

### 4.14 Scaphoid fractures

There are few studies on the morphology of scaphoid fractures. Scaphoid fractures are traditionally described by X-ray. Turow et al. (2020) made a 2D fracture map of scaphoid fracture based on the 3D CT data of 75 cases of scaphoid fractures. They found that lumbar fractures had a higher rate of comminution and displacement, with comminution located in the dorsal ridge and the volar scaphoid waist (Turow et al., 2020).

### 4.15 Talus fractures

Talus fractures account for 0.5% of all fractures and 3% of all ankle fractures (Adelaar, 1997; Sautet et al., 2021). Talar fractures are rare, but their complex anatomical structures lead to a high incidence of post-traumatic arthritis, joint stiffness, nonunion and osteonecrosis (Buza and Leucht, 2018). In 2024, Wang et al. (2024) reported the morphological characteristics of talus fractures in 126 cases. They found that the fracture line of talus fracture was mainly concentrated in the talus neck and lateral process of talus (Wang et al., 2024). In addition, they also suggested surgical approaches for different talus fractures.

### 4.16 Clavicular fracture

Chao et al. (2023) characterized the morphological characteristics of the fracture edge of clavicular shaft fracture. They found that the diaphyseal clavicular fractures had a coronal fracture edge, and the fracture was mainly located in the distal half of the diaphyseal segment.

## 5 Discussion

Fracture Mapping is a new technology developed in recent years that can clearly and intuitively display the distribution and morphology of fracture lines. It is widely used for various fracture, offering a novel approach for fracture classification, treatment plan selection, internal fixation design, fracture-prone area statistics, and the development of standardized fracture models. This review includes 74 studies, detailing various methods of fracture mapping and discussing existing issues within the technology, thus promoting advancements towards more precise mapping techniques. Additionally, this review systematically categorizes the included studies by fracture site, summarizing the morphological characteristics of fractures in various regions, which is crucial for determining optimal surgical methods and fixation devices.

Since 2009, when the orthopedic team at the University of Minnesota first introduced the concept of fracture mapping, there have been significant advancements in imaging technology and computational methods, greatly enhancing the accuracy and practicality of fracture mapping. Over the past decade, fracture mapping has evolved from 2D to 3D, from non-reconstruction to reconstruction, and from non-reduction to reduction. In some studies, fractures were not reduced during the mapping process. The non-reduced fracture model is challenging to accurately overlap with the standard model, which results in the fracture line not being accurately transcribed onto the standard template. A study conducted by Dugarte et al. (Dugarte et al., 2018) compared the accuracy of 2D fracture mapping with that of 3D fracture mapping. They found that 3D mapping demonstrated greater accuracy in comparison to 2D fracture mapping techniques. The implementation of 3D mapping will facilitate a better understanding of the 3D morphology of fractures. Instead, the fracture lines were transcribed onto 2D standard models based on their relationship with anatomical landmarks. Ensuring the accuracy of the position and morphology of fracture lines during transcription was challenging. Moreover, in some studies, researchers used CT cross-sections below the joint surface to draw fracture lines, which often fails to represent the true morphology of the fracture lines. Despite these shortcomings, these studies provided early reports on the morphology of these fractures, giving surgeons an initial understanding of their shapes. Compared to 2D mapping, 3D mapping offers a comprehensive view of the fracture site, allowing surgeons to observe the morphology of fracture lines from multiple angles. It also provides detailed visualization of complex fractures, enabling more accurate assessment than traditional 2D radiographs. This is a significant advantage over the limited perspective of 2D mapping. Additionally, 3D images can reveal the complex structure of fractures, including the precise location and arrangement of bone fragments, which are often difficult to discern in 2D images.

The lack of uniformity in fracture classification makes it difficult for physicians to communicate about the fracture morphology. An effective fracture classification system should diagnose the fracture, guide treatment, and predict the prognosis. Additionally, the repeatability and ease of use of the classification system are important, as it must be easily understood and applied by clinicians while yielding consistent results across different practitioners. Fracture mapping provides a clear visualization of fracture morphology and common sites, offering a new method for proposing fracture classifications. Among the literature included in this study, 11 studies have proposed new fracture classification systems (Table 11).

**TABLE 11:** Details of fracture classification.

Study	Fractures		Classification details		
A3:A14 (Zeng et al., 2022)	Ankle fractures		Fibula fractures and tibia fractures are divided into 3 and 4 types respectively, and combined according to the area involved		
			Fibula		a. Located under the joint tibiofibular ligament b. Located above the tibiofibular joint ligament c. Located on the joint tibiofibular ligament
			Tibia		A. Over the medial malleolar joint surface. B. Surrounding the Volkmann’s tuberosity in a ring shape. C. Similar to type B, but the annular fracture line was larger in diameter D. The fracture line run longitudinally from the subtalar articular surface up through the medial or posterior malleolus
Ren et al. (2023a)	Intertrochanteric fracture		I: The simple greater tuberosity + lesser tuberosity + medial cortex. II: The greater tuberosity + isolated fragment Lesser Tuberosity + Medial Cortex. III: The Greater Tuberosity + Lesser Tuberosity + Medial Isolated Fragment. IV: The Isolated Greater Tuberosity. V: The Greater Tuberosity + Lesser Tuberosity. VI: The Greater Tuberosity + Medial Cortex		
Yao et al. (2022b)	Hyperextension tibial plateau fracture		According to the distribution of fracture lines, hyperextension tibial plateau fractures are divided into 4 rows and 9 columns (Figure 8G)		
Ju et al. (2023)	Greater tuberosity fractures		They classified greater tuberosity fractures into five types: anterior-split, posterior-split, complete-split, anterior-avulsion, and posterior-avulsion types		
Li et al. (2019)	Intertrochanteric fracture		The head, greater trochanter, lesser trochanter, and shaft are considered as separate parts, and fractures are classified as two, three, or four part fractures based on their combination		
He et al. (2022)	Avulsion fractures of the fifth metatarsal base		I: Proximal fracture lines predominantly involves the action of the lateral band of the plantar fascia. II: Middle fracture lines predominantly involves the action of the peroneus brevis. IIIA: Distal fracture lines involves the joint action of the peroneus brevis and the lateral band of the plantar fascia, with only one fracture line. IIIB: Involves the joint action of the peroneus brevis muscle and the lateral band of the plantar fascia, with two fracture lines		
Wu et al. (2022)	Femoral head fractures		A: The superior-lateral part of the femoral head. B: The central part of the femoral head. B1: The fracture line limited to the femoral head and parallel to the axis of the primary compressive trabeculae. B2: The fracture line limited to the femoral head and unparallel to the axis of the primary compressive trabeculae. B3: The fracture line not limited to the femoral head or extended to other parts in the proximal femur. C: The anterior-inferior part of the femoral head		
E(Ren et al., 2023b)	Posterior-medial intertrochanteric fracture		I: Posterolateral + posteromedial + isolated fragment medial. II: Posterolateral + posteromedial + simple medial. IV: Isolated medial. V: Posteromedial + medial. VI: Isolated posterolateral		
Yang et al. (2018)	Quadrilateral plate fractures		The line between the ischial spine and the iliopectineal eminence divides the quadrilateral plate into two parts: posterior (Area “A”) and anterior (Area “B”) (Figure 7B). Correspondingly, fractures in Area “A” are defined as Type A fractures, fractures involving Area “B” are defined as Type B fractures, and fractures spanning both areas (Area “A + B”) are defined as Type C fractures		
(Zhang et al. (2019)	Intra-articular distal radius fractures		The fracture line distribution could be divided into five categories: “┴”, “┬”, “┤”, “├”, and “┼”		
Molenaars et al. (2015)	Tibial Plateau Fracture		(1) The lateral split fragment with or without comminution. (2) The posteromedial fragment. (3) The tibial tubercle fragment. (4) Comminuted fracture involving the tibial spine		

The fracture classification proposed based on fracture mapping has several characteristics: 1) It often performs better in terms of repeataibility, allowing for rapid diagnosis based on fracture images; 2) Compared to existing classifications, the classification based on fracture mapping is more comprehensive and can describe more types of fractures; 3) It can reflect critical morphological details and the condition of surrounding soft tissue damage; 4) It fills the gaps in the classification of certain special fractures, such as hyperextension tibial plateau fractures and avulsion fractures of the base of the fifth metatarsal. (Li et al., 2019; Zhang et al., 2019; He et al., 2022; Wu et al., 2022; Yao X. et al., 2022; Ju et al., 2023; Ren et al., 2023a).

Fracture mapping using CT helps us understand the most common displacements of fractures and determine the best approach for treating these injuries. Effective preoperative planning allows for the selection of optimal implants and their best position, ensuring fragments stability and achieving good outcomes. The study of Hasan et al. (2017) indicates that proximal humerus fractures rarely involve the intertubercular groove. Furthermore, due to the bicipital groove being an easily identifiable landmark, it can serve as a “safe zone” for suturing or the placement of screws. Ju et al. (2023) discovered that in fractures of the humeral greater tuberosity, anterior avulsion fragments and posterior fragments are usually not covered by the locking plate. These fragments tend to migrate after locking plate fixation, making additional fixation techniques essential to stabilize these greater tuberosity fracture fragments. Ye et al. (Ye et al., 2023a; Ye et al., 2023b) recommend using the Kocher-Langenbeck (K-L) approach for posterior and posterolateral T-type acetabular fractures because the fracture lines are mainly located in the posterior acetabulum and often associated with posterior wall fractures. However, for anterior fractures, the lines are concentrated very low in the anterior column, contraindicating a posterior approach; thus, they suggest an anterior approach. Oguzkaya et al. research found that acetabular both-column fractures combined with posterior wall fractures mainly affect the anterior acetabular stress. They recommend using a single ilioinguinal or iliac fossa combined with a Stoppa approach for treatment. For acetabular fractures with fracture lines concentrated medially, using a modified Stoppa approach may be beneficial (Oguzkaya et al., 2023b). Xiaohua et al. (McGonagle et al., 2019) suggest that most posteromedial tibial plateau fragments exhibit a mediolateral fracture line direction, which may lead to insufficient stability when inserting screws through an anterolateral plate. Patients with these fracture characteristics may benefit from additional medial or posterior surgical approaches. Su et al. (2020) reported on the fracture morphology of posterior malleolar fractures. They suggest using a posterolateral approach for supination-external rotation type IV injuries for fracture reduction and fixation. For pronation-external rotation type IV injuries, satisfactory reduction and stabilization can be achieved by placing screws in an anteroposterior direction. Shi et al. (2023) found that the most concentrated fracture lines in calcaneal fractures are slightly below the sinus tarsi approach. This suggests that screw placement should avoid this area when fixing from the lateral wall and indicates that a lower surgical approach may be more advantageous.

**FIGURE 9:**
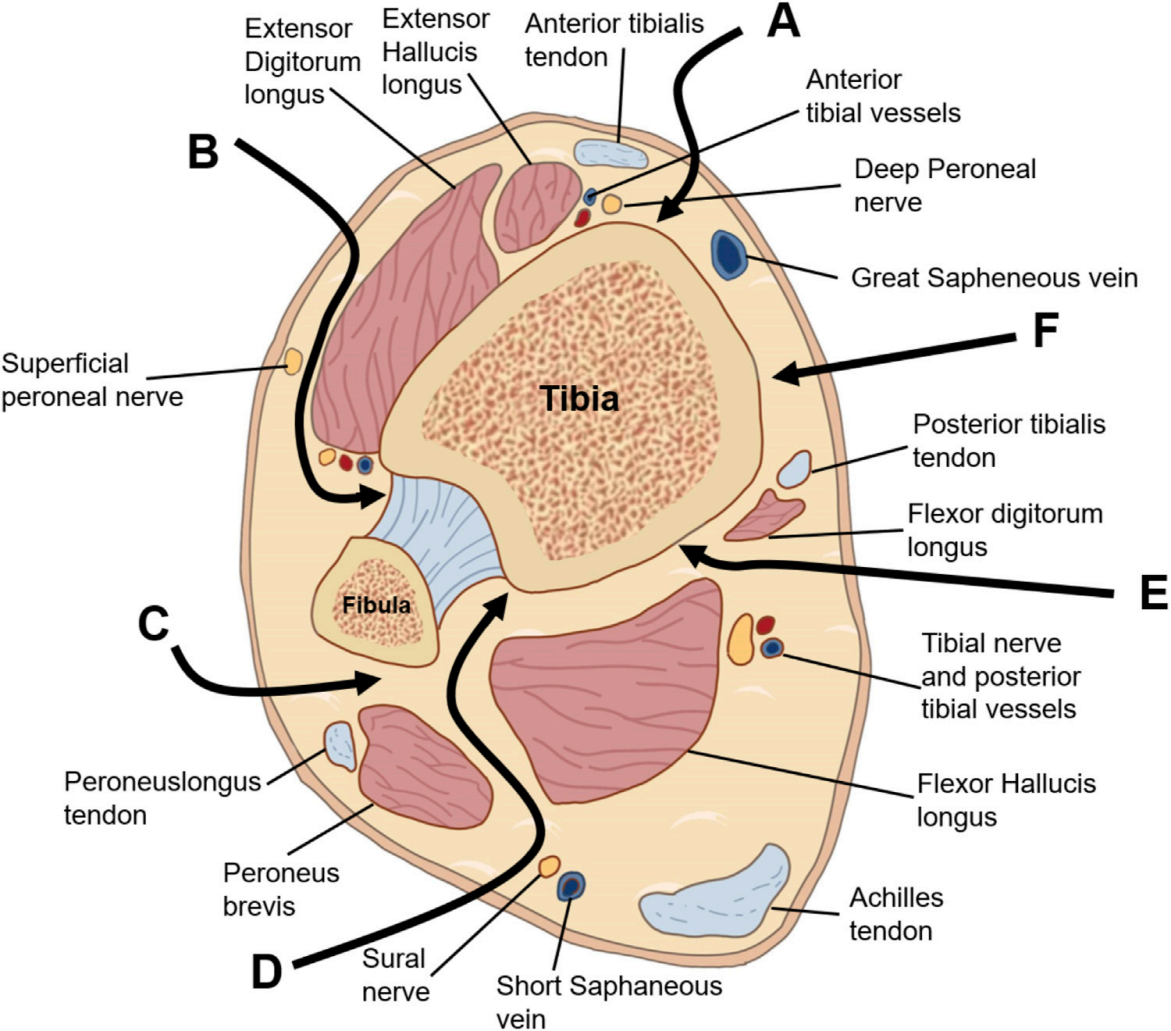
Depicts the intervals for standard surgical approaches to pilon fractures. **(A)** Anteromedial, **(B)** anterolateral, **(C)** direct medial, **(D)** posteromedial, **(E)** posterolateral fibula, **(F)** posterolateral tibia.

The morphology of Pilon fracture provides a reference for the choice of surgical approach. The surgical approach to the distal tibia is illustrated in Figure 9. For C1.1 fractures (no impaction) and C1.3 fractures (extending to the diaphysis), the location of the implant depends on the height of the fracture line, and the anterolateral or medial approach is preferred (Cole et al., 2013). For C1.2 fractures (with impaction), it is recommended to use the anteromedial approach to place the anterior locking plate. The fracture fragments of C2 Pilon fracture are mainly concentrated in the anteromedial and anterolateral distal tibia (Cole et al., 2013). Therefore, it is recommended that the implant be placed in a position covering multiple fragments through the anteromedial approach or the anterolateral approach. For C3 fractures, extensive involvement of the distal articular surface of the tibia requires fixation of major fragments (anterior, medial, and posterior) as well as small fragments (anterolateral and posterolateral). The current implant screw trajectory may miss the medial, anterolateral, and Volkmann fragments. The anatomical distal tibial locking plate may not be able to fully fix the anterolateral fracture piece in some sizes (Sohn et al., 2018). Therefore, C3 fractures should be treated with a combined surgical approach (Zelle et al., 2019).

Bone biomechanics is one of the many branches of biomechanics (Jabran et al., 2019). It mainly applies biomechanical principles and engineering technology to study, prevent and treat fractures. Biomechanical studies of fractures can compare different implants and different surgical methods, thereby providing a reference for surgeons (Varga et al., 2017). The establishment of fracture models is a crucial component of biomechanical research. Different osteotomy positions and osteotomy gaps may produce different results. At present, there is no standard fracture model for most fractures. The fracture map can intuitively show the common location of the fracture line, providing a reference for the establishment of a standard biomechanical fracture model. Based on the distribution frequency of fracture lines, Li et al. (Li R. et al., 2022) suggest that for AO/OTA 33A distal femoral fracture models, the osteotomy gap should be approximately 4 cm proximal to the joint line, which aligns with Chen et al. (2023). Li et al. recommend an osteotomy gap of 4 cm, while Chen et al. suggest 6 cm. For the 33C distal femoral fracture model, Li et al. recommend a supracondylar osteotomy 5 cm proximal to the joint line with a 4 cm osteotomy gap, whereas Chen et al. (Chen et al., 2023) suggest starting the osteotomy 3 cm from the joint line with a 4 cm gap. The fracture models proposed by both studies are similar. The resulting distal femoral fracture models may be more accurate and realistic than those generated in previous studies. Other fracture mapping studies have not provided osteotomy recommendations for fracture models, which needs to be addressed in future research.

However, existing studies on fracture maps also have some limitations: 1) The sample sizes of included fracture cases are relatively small. Seven studies included fewer than 50 patients (7/74), and 33 studies included fewer than 100 patients (33/74). With such small sample sizes, the results may be subject to significant variability, and the fracture maps may not adequately represent the fracture characteristics of these patients. 2) The fracture mapping process is time-consuming and costly. Advances in software algorithms, artificial intelligence (AI), integration with machine learning or integration with finite element modeling are expected to improve the accuracy and automation of fracture mapping, significantly improving research efficiency (Adouni et al., 2019; Faisal et al., 2019; Adouni et al., 2020). 3) Current mapping studies are mostly limited to establishing fracture classifications. There is a lack of research on whether these classifications can assess fracture prognosis or guide treatment, limiting their clinical application. Future research should focus on the potential of mapping to guide surgical approaches and the design of internal fixation devices. 4) All studies are retrospective, which may introduce selection bias in patient inclusion. 5) Potential inaccuracies in 3D model reconstruction and software compatibility issues can affect the accuracy of fracture mapping.

## 6 Conclusion

Fracture mapping is a new technology developed in recent years. Currently, it has been widely used in acetabular fractures, scapular fractures, proximal humerus fractures, Pilon fractures, etc. We reviewed 76 studies, detailing various methods for fracture mapping and highlighting existing issues, in order to provide a reference for future fracture mapping research. Additionally, we also summarized the morphology of various fractures to enhance orthopedic surgeons’ understanding of the characteristics of these fractures.
